# Apolipoprotein E Deficiency Exacerbates Spinal Cord Injury in Mice: Inflammatory Response and Oxidative Stress Mediated by NF-κB Signaling Pathway

**DOI:** 10.3389/fncel.2018.00142

**Published:** 2018-05-23

**Authors:** Xuan Yang, Shurui Chen, Zhenya Shao, Yuanlong Li, He Wu, Xian Li, Liang Mao, Zipeng Zhou, Liangjie Bai, Xifan Mei, Chang Liu

**Affiliations:** ^1^School of Nursing, Jinzhou Medical University, Jinzhou, China; ^2^Department of Orthopedics, First Affiliated Hospital of Jinzhou Medical University, Jinzhou, China; ^3^Department of Endocrinology, First Affiliated Hospital of Jinzhou Medical University, Jinzhou, China; ^4^Department of Orthopedics, Third Affiliated Hospital of Jinzhou Medical University, Jinzhou, China; ^5^Department of Oncology, First Affiliated Hospital of Jinzhou Medical University, Jinzhou, China; ^6^Department of Orthopedics, First Affiliated Hospital of China Medical University, Shenyang, China

**Keywords:** contusive spinal cord injury, Apolipoprotein E, neuroinflammation, oxidative stress, apoptosis

## Abstract

Spinal cord injury (SCI) is a severe neurological trauma that involves complex pathological processes. Inflammatory response and oxidative stress are prevalent during the second injury and can influence the functional recovery of SCI. Specially, Apolipoprotein E (APOE) induces neuronal repair and nerve regeneration, and the deficiency of *Apoe* impairs spinal cord-blood-barrier and reduces functional recovery after SCI. However, the mechanism by which *Apoe* mediates signaling pathways of inflammatory response and oxidative stress in SCI remains largely elusive. This study was designed to investigate the signaling pathways that regulate *Apoe* deficiency-dependent inflammatory response and oxidative stress in the acute stage of SCI. In the present study, *Apoe*^−/−^ mice retarded functional recovery and had a larger lesion size when compared to wild-type mice after SCI. Moreover, deficiency of *Apoe* induced an exaggerated inflammatory response by increasing expression of interleukin-6 (IL-6) and interleukin-1β (IL-1β), and increased oxidative stress by reducing expression of Nrf2 and HO-1. Furthermore, lack of *Apoe* promoted neuronal apoptosis and decreased neuronal numbers in the anterior horn of the spinal cord after SCI. Mechanistically, we found that the absence of *Apoe* increased inflammation and oxidative stress through activation of NF-κB after SCI. In contrast, an inhibitor of nuclear factor-κB (NF-κB; Pyrrolidine dithiocarbamate) alleviates these changes. Collectively, these results indicate that a critical role for activation of NF-κB in regulating *Apoe*-deficiency dependent inflammation and oxidative stress is detrimental to recovery after SCI.

## Introduction

Spinal cord injury (SCI) can lead to destructive and irreversible neurological deficits, including sensory, motor and autonomic impairments (Lang et al., 2015). The complex pathological process that occurs after SCI is biphasic, consisting of a primary injury followed by the spreading of secondary tissue damage (Bartus et al., 2016). The primary injury directly induces irreversible cell death at the initial traumatic site. In contrast, the secondary injury is characterized by inflammatory responses, oxidative damage, neuronal death and other molecular events, which can be alleviated (Oyinbo, 2011). Currently, treatment of SCI includes early interventions in the acute stage aimed at minimizing secondary complications and preventing the spread of damage beyond the primary injured site (Ramer et al., 2014). Numerous studies have also indicated that targeting this secondary damage induced by cytokines and signaling pathways could be a new therapeutic strategy to help improve neurological outcomes after SCI (Coll-Miró et al., 2016; Zheng et al., 2017). Because inflammation, oxidative stress and apoptosis play contributory roles in the pathogenesis of the secondary damage, it is indispensable and important to explore novel methods of inhibiting secondary injury as treatment strategies for patients with SCI.

Inflammatory and oxidative responses are two major components of the secondary injury and play fundamental roles in the pathogenesis of SCI (Silva et al., 2014; Anwar et al., 2016). NF-κB, a redox sensitive transcription factor, plays a vital regulatory role in inflammatory responses after SCI (Bethea et al., 1998; Rafati et al., 2008). Activation of NF-κB involved in the occurrence of SCI is necessary for expression of inflammatory factors, such as interleukin-6 (IL-6), tumor necrosis factor-α (TNF-α), interleukin-1β (IL-1β; Lu et al., 2013). Nuclear factor erythroid-derived 2-related factor 2 (Nrf2) is accountable for anti-oxidative responses and anti-inflammatory properties in neurological disorders (Kanninen et al., 2015; Wang et al., 2017). After SCI, Nrf2 is up-regulation and activates a group of anti-oxidative and cytoprotective genes, such as heme oxygenase-1 (HO-1) and NADPH quinineoxidoreductase-1 (NQO1; Pomeshchik et al., 2014). Previous study shown that Nrf2 regulates the inflammation by limiting the up-regulation of NF-κB activity after SCI (Mao et al., 2010). Thus, inactivation of NF-κB and induction of the Nrf2 pathway in SCI might be an effective neuroprotective strategy.

Apolipoprotein E (APOE: protein, *Apoe*: gene), mainly synthesized in astrocytes of central nervous system (CNS), is involved in lipid metabolism and cholesterol transport for the promotion of neuronal repair (Bu, 2010; Mahley and Huang, 2012). It is well-established that *APOE4* is the strongest genetic risk factor for early onset AD among its other three alleles (2, 3, and 4; Liu et al., 2017; Nuriel et al., 2017). Previous studies show APOE is up-regulated *in vivo* after neurological dysfunction, such as SCI (Seitz et al., 2003), traumatic brain injury (Iwata et al., 2005), chronic stroke (Kim et al., 2015), chronic constriction injury (Bellei et al., 2017). More specifically, *Apoe* possesses anti-inflammatory, anti-oxidative and anti-apoptosis properties (Laskowitz et al., 1997, 1998; Kitagawa et al., 2002; Hayashi et al., 2009; Li X.-Z. et al., 2017). For instance, APOE mimetic peptide significantly suppressed microglial activation in rats with SCI. Conversely, the excessive inflammation may be caused by the increased permeability of BSCB leading to the influx of inflammatory cells in peripheral blood in *Apoe*^−/−^ mice after SCI (Wang et al., 2014; Cheng et al., 2018). Moreover, *Apoe*^−/−^ mice showed that increased oxidative stress caused an exaggerated neuronal deposition and contributed to the neurodegenerative processes (Nuriel et al., 2017). Also, APOE plays a critical role in the clearance of apoptotic bodies during neuronal apoptosis (Elliott et al., 2007). Further studies revealed various signaling pathways were involved in the anti-inflammatory, anti-oxidative stress and anti-apoptosis role of APOE. For example, depletion of *Apoe* caused abnormal cerebrovascular and neuronal function by activating a proinflammatory CypA–NF-κB–MMP9 pathway (Bell et al., 2014). Also, extracellular APOE interacts with its receptor to activate mitogen-activated protein kinase (MAPK) signaling pathway to regulate inflammation and apoptosis (Pocivavsek and Rebeck, 2009; Gan et al., 2011; Safina et al., 2016).

Although recent studies shown APOE has a critical neuroprotective function after SCI, the molecular mechanism of *Apoe* interaction with NF-κB pathway in the alleviation of inflammatory response and oxidative stress of SCI remains unknown. Here, we demonstrated that deficiency of *Apoe* retarded the recovery of locomotor function and tissue repair via increasing the expression of inflammatory cytokines and oxidative response, as well as aggravating neural apoptosis of the spinal cord tissue *in vivo*. In addition, we further showed *Apoe* deficiency caused inflammation and oxidative stress could be alleviated through inhibition of NF-κB signaling pathway.

## Materials and Methods

The present study was conducted using female *Apoe*^−/−^ mice with a C57BL/6J background (strain B6/JNju-*Apoe*em1Cd82/Nju; stock number T001458), weighing 20–25 g, and 10–12 weeks. *Apoe*^−/−^ mice were purchased from the Nanjing biomedical Research Institute of Nanjing University and have been described previously (Gallardo et al., 2008). Homozygous (ApoE^−/−^) mice were completely Knockout *Apoe* were verified by PCR. The age-matched, sex-matched littermates of *Apoe*^−/−^ mice or WT controls were used for western blot (WB), immunofluorescence staining, as well as histological staining. All mice were housed in standard cages (five per cage) in a specific pathogen free (SPF) laboratory animal center of Jinzhou Medical University. They were given free access to food and water *ad libitum*, under a 12 h/12 h light/dark cycle. The Institutional Animal Care and Use Committee of Jinzhou Medical University approved all experimental protocols (permit number: SCXK (Liao) 2014-0004).

### Experimental Design

Mice were randomly allocated to the following four parts of the experiment using a completely random number table.

### Part 1

A total of 48 WT mice were randomly divided into eight experimental groups, including sham (Sham operation after 7 days), 12 h, 1 day, 3 days, 5 days, 7 days, 14 days and 28 days, to evaluate temporal changes of APOE protein levels after SCI.

### Part 2

WT (*n* = 6) and *Apoe* KO (*n* = 6) mice were used to evaluate the effect of *Apoe* deficiency on behavioral recovery, which was observed from before and after SCI days 1–28.

### Part 3

Sham (*n* = 6) and SCI (*n* = 6) group in WT and *Apoe* KO mice were sacrificed at 7 dpi for WB. The same numbers of WT and *Apoe* KO mice were sacrificed for histological analysis. These experimental mice were randomly assigned to four groups: the WT-Sham group, WT-SCI group, KO-Sham group and KO-SCI group.

### Part 4

WT (*n* = 24) and *Apoe* KO (*n* = 24) mice were randomly divided into four groups: WT-SCI and vehicle-treated group (WT-SCI-Veh), KO-SCI and vehicle-treated group (KO-SCI-Veh), WT-SCI and PDTC-treated (WT-SCI-PDTC), KO-SCI and PDTC-treated group (KO-SCI-PDTC). PDTC groups were intraperitoneal injection with an inhibitor of NF-κB, PDTC (P8765, Sigma-Aldrich, Darmstadt, Germany) 100 mg/kg/day as previously described (Jiménez-Garza et al., 2005; Bell et al., 2014). While the vehicle groups were injected with the equivalent volume of saline after 5 min SCI and then seven consecutive days.

### Spinal Cord Contusion Injury Model

All experimental interventions were carried out in accordance with the Guidelines for Animal Experiments provided by our institution. A mouse model of SCI was established using the modified weight-drop method (Ma et al., 2001). The mice were anesthetized with 10% chloral hydrate (0.33 mL/kg, intraperitoneal injection) until they were confirmed to be unconscious by toe-pinch. After immobilizing the vertebrae with a spinal stereotactic device, the skin and muscles were incised along the dorsal midline to expose the vertebral column, and laminectomy was performed at the T10 level. A 3 g-impactor device (diameter: 0.5 mm) was subsequently dropped from a height of 25 mm to the spinal cord to induce immediate hind limb paralysis. After SCI, the muscles and skin aseptically sutured in layers. Sham animals only received laminectomy. A manual bladder emptying was performed three times daily until the urinary emptying function was re-established in mice after SCI.

### Western Blot Analysis

Mice were sacrificed at 7 days after SCI and injured spinal cord tissues (0.5 cm length from the injury epicenter) were rapidly extracted for WB. The spinal cord tissue was homogenized in RIPA lysis buffer containing PMSF buffer (P0013B, Beyotime, Beijing, China) for 30 min on ice. After centrifugation at 12,000 RMP (25 min, 4°C) to remove debris, the supernatant was quickly stored at −80°C. The BCA Protein Assay Kit (P0010, Beyotime, Beijing, China) was used to quantify protein levels. Protein (40 μg) was loaded at 10% or 12% SDS-PAGE. Following electrophoresis, the protein was transferred to PVDF membranes (0.22 μm, Merckmillipore, Darmstadt, Germany). The membranes were blocked with 5% dried skimmed milk in TBS with 0.1% Tween-20 for 2 h at RT, and then incubated at 4°C overnight with the following primary antibodies: mouse anti-GAPDH (1:10,000, HC301-01, Transgene, Beijing, China), rabbit anti-APOE (1:200, sc-13520, Santa Cruz Biotechnology, CA, USA), mouse anti-GFAP (1:1000, ab7260, Abcam, Cambridge, England), mouse anti-IL-6 (1:1000, ab9324, Abcam, Cambridge, England), rabbit anti-IL-1β (1:1000, ab9722, Abcam Cambridge, England), rabbit anti-Bcl-2 (1:1000, ab136285, Abcam, Cambridge, England), rabbit anti-Bax (1:1000, ab32503, Abcam, Cambridge, England), rabbit anti-Nrf2 (1:1000, ab31163, Abcam, Cambridge, England), rabbit anti-p-NF-κB-p65 (1:1000, ab86299, Abcam, Cambridge, England), mouse anti-HO-1 (1:1000, ab3248, Abcam, Cambridge, England), mouse anti-NQO-1(1:1000, ab2947, Abcam, Cambridge, England), mouse anti-Erk1(pT202/pY204) + Erk2(Pt185/Py187; 1:1000, 50011, Abcam, Cambridge, England), rabbit anti-ERK1 + ERK2 (1:1000, 184699, Abcam, Cambridge, England), rabbit anti-Phosphp-p38-MAPK(Thr180/Tyr182; 1:1000, 9211, Cell Signaling Technology, Danvers, MA, USA), rabbit anti-p38-MAPK (1:1000, 9212, Cell Signaling Technology, Danvers, MA, USA), and rabbit anti-JNK1 (pThr183/pThy185; 1:1000, NBP1-72242, Novus Biologicals, Littleton, CO, USA). The membranes were then incubated with the corresponding secondary antibodies for 2 h at RT. Subsequently, the membranes were visualized using a ChemiDoc-ItTMTS2 Imager (UVP, CA, USA). Finally, a blinded researcher analyzed the bands using the ImageJ2x software program (National Institute of Health, New York, NY, USA).

### Tissue Processing

The mice were sacrificed with an overdose anesthetic and transcardially perfused with 0.9% saline, followed by 4% PFA in PBS at 7 days after injury. Spinal cord tissues containing areas rostral and caudal to the epicenter of injury (about 2 cm) were harvested and fixed in 4% PFA overnight at 4°C. Tissue segments then dehydrated in 30% sucrose-PFA until they sunk close to the bottom of the liquid. Finally, the spinal cord tissues (lesion epicenter about 2 cm) were cut with a cryostat microtome (CM3050S, Leica, Wetzlar, Germany) into 10 μm-thick longitudinal and 20 μm-thick transverse serial sections for immunofluorescence, Hematoxylin & Eosin (H&E) staining and Nissl staining.

### Immunofluorescence Staining

Briefly, the spinal cord sections were incubated overnight at 4°C with primary antibodies. The following primary antibodies were used for immunofluorescence: rabbit anti-APOE (1:200, sc-13520, Santa Cruz Biotechnology, Santa Cruz, CA, USA), mouse anti-NeuN (1:500, 104224, Abcam, Cambridge, England), rabbit anti-GFAP antibody (1:200; DAKO, Glostrup, Denmark), mouse anti-GFAP (1:1000, ab7260, Abcam, Cambridge, England), mouse anti-CD68 (1:500, NBP2-33337, Novus, Plymouth, MN, USA) and rabbit anti-Cleaved-caspase-3 (1:200, 9664, Cell Signaling Technology, Danvers, MA, USA). On the following day, the sections were rinsed and incubated with appropriate secondary antibody, including goat anti-mouse IgG (H + L) cross-adsorbed secondary antibody, Alexa Fluor^®^ 488 (1:400, A-11017, Invitrogen, Carlsbad, CA, USA) and goat anti-rabbit IgG (H + L) cross-adsorbed secondary antibody, Alexa Fluor^®^ 568 (1:400, A-11019, Invitrogen, Carlsbad, CA, USA) for 2 h at RT. Finally, the nuclei were counterstained with 4′,6-diamidino-2-phenylindole (DAPI, 1:1000, 104139, Abcam, Cambridge, England) for 15 min at RT. All sections were mounted on Permount™ mounting medium before being screened using a fluorescence microscope (DMI4000B, Leica, Wetzlar, Germany).

### Behavioral Assessments

Hind limb motor function was monitored by the Basso Mouse Scale (BMS) test at 1 day before SCI and then weekly for 4 weeks (Basso et al., 2006). Two raters who were blinded to the experimental groups collected and analyzed the data. Briefly, each mouse was observed for 4 min, and hind limb movement performance was assessed and corresponded to the nine-point scales. The final score of each animal was the mean values from both investigators who were blinded to the grouping and experimental design.

### Hematoxylin and Eosin (H&E) Staining

The spinal cord longitudinal sections were taken at 7 days after SCI and were immersed in hematoxylin solution for 3 min, following by rinsing distilled water quickly. Then the slides were differentiated in HCL/95% alcohol (1:50) solution for 10 s. After washing with distilled water for 5 min, the sections were stained with 0.5% eosin for 10 s. After dehydration used by gradient ethanol for 3 min (95%, 100%), and then transparency by xylene for 1 min. Next, the maximum area of the lesion epicenter in each section was examined with a light microscope (DMI4000B, Leica, Wetzlar, Germany).

### Nissl Staining

In accordance with the manufacturer’s instructions of Nissl staining, the sections of spinal cord incubated in 0.1% Nissl staining solution (C0117, Beyotime, Beijing, China) for 3 min at 37°C, rinsed with distilled water, dehydrated in by 95% and 100% ethanol solutions for 2 min, respectively, and cleared in xylene for 5 min. The Nissl-stained images were captured on two sides of each section in the ventral horn at under an optical microscope (DMI4000B, Leica, Wetzlar, Germany).

### Quantitative Image Analysis

For quantification of the lesion area, we performed as previously described (Cheng et al., 2016; Narang et al., 2017), the spinal cord segment of 0.5 cm contains the epicenter of the lesion. The lesion area was defined as the areas without normal tissue architecture, or tissue containing only cellular debris, which it showed a different optical density compared to the adjacent tissue. The border of the damage tissue was manually outlined with a dashed line. Four representative sections per animal (one of every 10 serial sections were stained), were traced three times manually outlined and analyzed by the ImageJ2x software program (National Institute of Health, New York, NY, USA). The extent of lesion expressed as percentage of lesion area per spinal cord on the same rostral-caudal axis was calculated in the sagittal plane. For quantification of the number of surviving neurons, transverse spinal cord sections were selected (four sections per animal) ranging from rostral (−300, −600 μm) to caudal (+300, +600 μm) around the epicenter. As the section thickness is 20 μm, it can be inferred that there are 15 sections in the 300 μm range. Neurons with a clear nucleolus and a Nissl bodies were counted by using previously published methods (Li et al., 1994; Li J. et al., 2017). According to Rexed’s lamina system of gray matter, the mean number of ventral motor neurons on two sides (lamina IX) was manually calculated through ImageJ2x software. For quantification of APOE-immunoreactive signal intensity, transverse spinal cord sections (four sections per animal) were selected ranging from rostral (−400, −700 μm) to caudal (+400, +700 μm) around the injury epicenter. An area of interest (ROI) was placed on the right and the left ventral horn of each section. Next, the APOE-immunoreactive optical density value was quantified by ImageJ2x software as described previously (Hong et al., 2017). For quantification of the NeuN/Cleaved-caspase-3 double-positive cells were performed as previously method (Bai et al., 2017), which were represented the positive of SCI-induced apoptotic cells. Four transverse sections were chosen from each animal in four position points, such as ±500 μm, ±800 μm. The optical density of double-labeled of Cleaved-caspase 3-positive neurons in the ventral motor regions was quantified using ImageJ2X. The double-staining of APOE and NeuN, GFAP and CD68 were selected ranging from rostral to caudal (±1000 μm) around the injury epicenter. All quantitative image analysis was conducted by two researchers blinded to the allocation.

### Statistical Analysis

Quantitative data are presented as mean ± SEM. Two experimental groups were analyzed by unpaired Student’s *t*-test. Comparisons of more than two groups were performed with a one-way analysis of variance (ANOVA) followed by Tukey’s *post hoc* test. The BMS scores were analyzed by repeated measures two-way analyses of variance (ANOVAs), followed by Tukey’s *post hoc* test to compare the differences between each group. Statistical analysis was performed using GraphPad Prism software, version 7.00 (GraphPad Software Inc., La Jolla, CA, USA). Significance was considered at *p* < 0.05.

## Results

### Expression of APOE in Normal and Injured Spinal Cord and *Apoe*^−/−^ Mice

**Figure 1:**
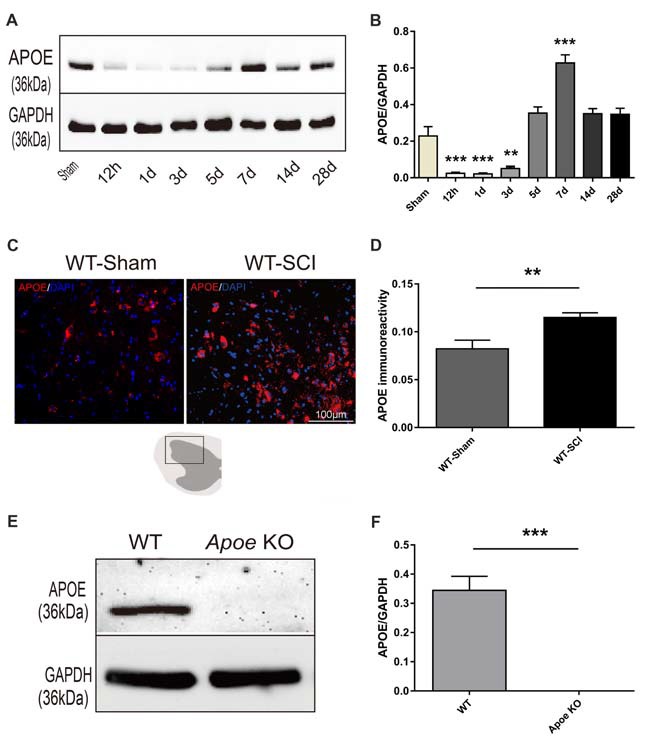
The temporal expression of Apolipoprotein E (APOE) protein after spinal cord injury (SCI) and identification of *Apoe* KO mice. **(A)** Representative western blots (WBs) of APOE and GAPDH expression in the lesion site after SCI. **(B)** Statistical analysis of WBs of APOE expression in WT mice after SCI. Data are expressed as mean ± SEM of three independent experiments (*n* = 6 per group). **p < 0.01, ***p < 0.001, Sham vs. 12 h, *p* = 0.0002, Sham vs. 1 day, *p* = 0.0001, Sham vs. 3 days, *p* = 0.0021, Sham vs. 7 days, *p* < 0.0001, one-way analysis of variance (ANOVA) followed by Tukey’s *post hoc* test. **(C)** Representative immunofluorescent images of APOE at the lesion epicenter in Sham and SCI group 7 days post-injury. **(D)** Statistical analysis of APOE immunoreactivity after SCI. Four transverse sections were measured optical intensity at ± 400 μm, ±700 μm from rostral and caudal to the injury site per spinal cord. Data are expressed as mean ± SEM of three independent experiments (*n* = 6 per group). **p < 0.01, Sham vs. 7 days, *p* = 0.0047, Student’s *t*-test, Scale bars = 100 μm. **(E)** APOE expression in the spinal cord was detected by WBs from WT and *Apoe* KO mice.** (F)** Statistical analysis of APOE and GAPDH expression both in WT and *Apoe* KO mice. Data are expressed as mean ± SEM of three independent experiments (*n* = 6 per group). ***p < 0.001, WT vs. *Apoe* KO, Student’s *t*-test.

WBs and immunofluorescence were used to examine the dynamic changes of APOE protein expression in sham and injured spinal cords of WT mice. Compared with the sham group, the expression of APOE began to decline in the first 3 days, but then increased on day 5. The highest level of APOE expression occurred 7 days post-injury (dpi; WT-Sham vs. WT-SCI: 0.23 ± 0.05 vs. 0.63 ± 0.04, *p* < 0.001, Figures 1A,B), and subsequently returned to uninjured levels 14–28 dpi (Figures 1A,B). In addition, increased APOE expression was observed 7 dpi using immunofluorescence (WT-Sham vs. WT-SCI: 0.08 ± 0.01 vs. 0.12 ± 0.01, *p* < 0.01, Figures 1C,D), which was in agreement with the WB data. We next used *Apoe* KO mice to explore the role of endogenous APOE in the pathology of SCI. WB was used to confirm that deletion of *Apoe* caused deficient APOE expression. As shown in Figures 1E,F, APOE expression is lost in spinal cords of *Apoe* KO compared with WT mice (WT-Sham vs. *Apoe* KO: 0.34 ± 0.05 vs. 0.00 ± 0.00, *p* < 0.001, Figures 1E,F). The other two representative WBs images for APOE proteins assessed are presented on Supplementary Figure S3.

### APOE Expression in Specific Cell Types of Normal and Injured Spinal Cord

**Figure 2:**
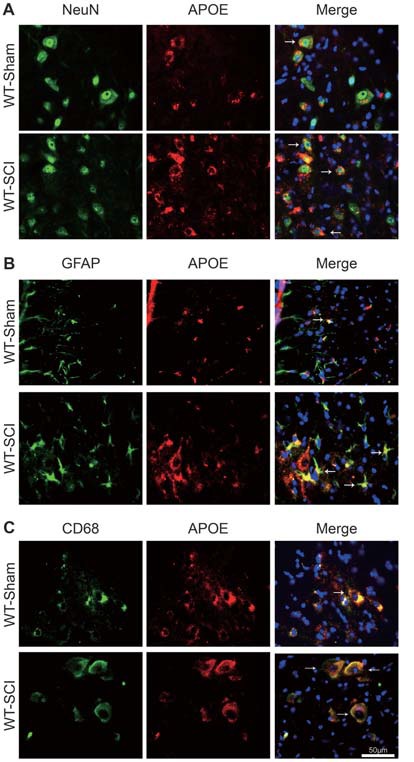
Expression of APOE protein in neurons, astrocytes, macrophages/microglia following SCI.** (A–C)** The cellular localization of APOE was observed by double immunofluorescent staining of APOE (red) with NeuN, GFAP, CD68 (green) and DAPI (blue), respectively. Four transverse sections were taken at ±1000 μm from rostral and caudal to the injury site per spinal cord (*N* = 6 per group, white arrows: APOE-positive neurons, astrocytes and macrophages, Scale bars = 100 μm).

To determine the expression pattern and possible cellular source of APOE, immunofluorescent co-localization of APOE with neurons (NeuN), astrocytes (GFAP), and macrophages (CD68) in uninjured and contused WT mice at 7 dpi was conducted (Figures 2A–C). As previously demonstrated, APOE was mostly distributed in astrocytes of the CNS, also in some neurons and macrophages under normal situation. It was obviously increased in these cells after CNS injury. We demonstrated that APOE was detectable in normal spinal cord of WT mice. At 7 dpi, APOE expression increased in the injured spinal cord, with high-level co-localization in the cytoplasm of numerous NeuN-positive neurons, GFAP-positive astrocytes and CD68-positive macrophages.

### *Apoe* Deficiency Impairs Functional Recovery and Histology After SCI

**Figure 3:**
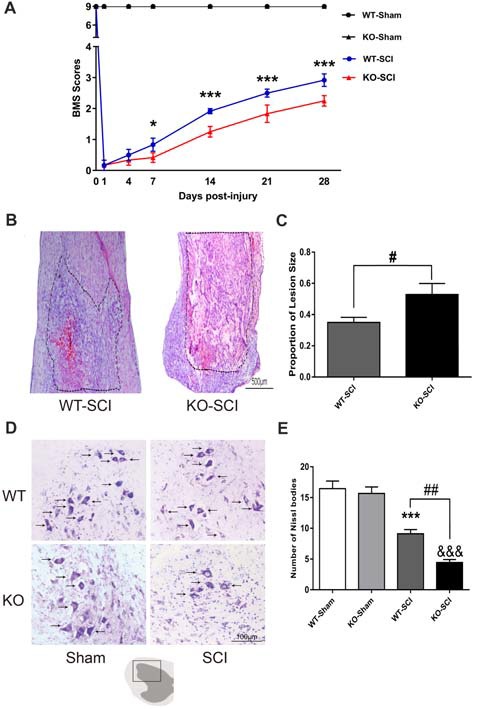
*Apoe* deficiency retards motor functional recovery and increases lesion area following SCI.** (A)** Statistical analysis of motor functional recovery of WT and *Apoe* KO mice before and after SCI using Basso Mouse Scale (BMS) scores. Data are expressed as mean ± SEM of seven independent experiments (*n* = 6 per group). *p < 0.05, ***p < 0.001, WT-SCI vs. KO-SCI, 7 days, *p* = 0.0493; 14 days, *p* = 0.0004; 21 days, *p* = 0.0004; 28 days, *p* = 0.0004, two-way ANOVA followed by Tukey’s *post hoc* test. **(B)** Representative micrograph of longitudinal spinal cord sections stained with H & E in both *Apoe* KO and WT mice at 7 days post-injury. The boundary of the lesion is outlined with a dotted line. **(C)** Quantitative analysis of the lesion area that determined from the length from injury site rostral and caudal 0.5 cm and expressed as percentage of lesion area per spinal cord analyzed. Four representative sections per animal (one of every ten serial sections) were stained. Data are expressed as mean ± SEM of three independent experiments (*n* = 6 per group). ^#^*p* < 0.05, WT-SCI vs. KO-SCI, *p* = 0.0392, Student’s *t*-test, Scale Bars = 500 μm. **(D)** Representative sections showing normal neurons that contained prominent nucleoli, loose chromatin, and Nissl body (arrow). **(E)** Quantitative analysis of motor neurons in the ventral horn at 7 days post-injury. Four transverse sections were chosen at ±300 μm, ±600 μm from rostral to caudal around the epicenter were obtained from each animal. Data are expressed as mean ± SEM of three independent experiments (*n* = 6 per group). ***p < 0.001, WT-Sham vs. WT-SCI, *p* < 0.001; ^&&&^*p* < 0.001, KO-Sham vs. KO-SCI, *p* < 0.001; ^##^*p* < 0.01, WT-SCI vs. KO-SCI, *p* = 0.0055, one-way ANOVA followed by Tukey’s *post hoc* test, Scale Bars = 100 μm.

Next, we evaluated the functional recovery of hind limb after SCI by BMS. There were not any differences in *Apoe* KO mice and WT mice before an injury, and then the movement function was abolished immediately on the first day after SCI. Already at 7 dpi, the BMS scores of *Apoe* KO mice were significantly reduced when compared to WT mice (WT-SCI vs. KO-SCI, 0.83 ± 0.21 vs. 0.42 ± 0.15, *p* < 0.05, Figure 3A), and this weakens in the locomotor performance of the *Apoe* KO mice for the subsequent 3 weeks period. Specifically, *Apoe* KO mice obtained an average BMS score of 2.25, which corresponds to a slight or extensive movement of the ankle following 28 dpi. On the contrary, an average BMS score of 2.92, WT mice showed extensive ankle movement and plantar paw placement without weight support (WT-SCI vs. KO-SCI, 2.92 ± 0.20 vs. 2.25 ± 0.17, *p* < 0.001, Figure 3A). These data clearly demonstrate that *Apoe* deficiency worsens the recovery of functional disabilities. Moreover, the lesion area evaluated by H&E staining in *Apoe* KO mice was greater than WT mice at 7 days after SCI (WT-SCI vs. KO-SCI, 0.53 ± 0.07 vs. 0.35 ± 0.03, *p* < 0.05, Figures 3B,C). *Apoe* KO mice exhibited less numbers of intact motor neurons and more damaged cells in the ventral horn of than in that of WT mice after 7 dpi (WT-SCI vs. KO-SCI, 9.11 ± 0.68 vs. 4.44 ± 0.47, *p* < 0.01, Figures 3D,E). Reactive astrogliosis are pathological markers of SCI. Glial scars can be used as a barrier to prevent regeneration after SCI. Therefore, we evaluated whether *Apoe*^−/−^ mice exhibit aggravated astrogliosis. The level of GFAP protein was performed by WBs and GFAP immunofluorescence staining was measured to assess the degree of astrogliosis at 1 mm caudal and 1 mm rostral to the epicenter of the lesion. No significant differences in the *Apoe* KO mice and WT mice at 7 dpi (Supplementary Figure S1). These results suggest that *Apoe* deficiency impedes the neurological function and histological outcomes after SCI.

### *Apoe* Deficiency Elevates Neuroinflammation After SCI

**Figure 4:**
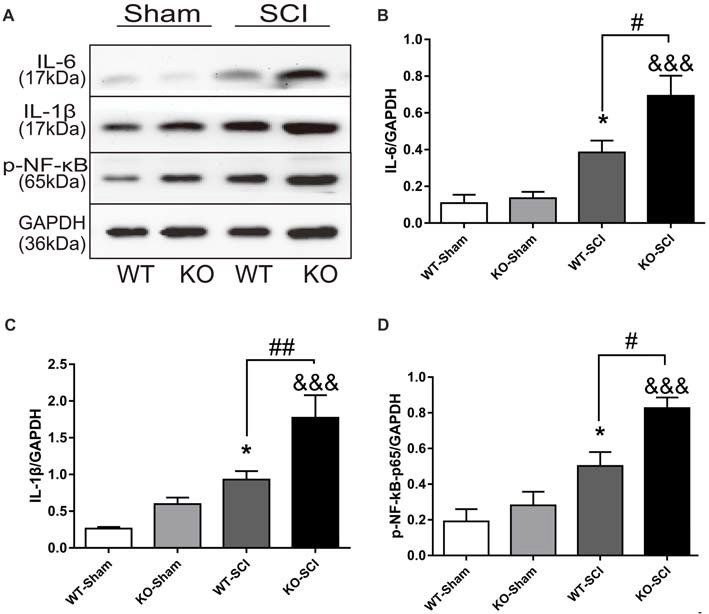
*Apoe* deficiency aggravates inflammatory response after SCI. **(A)** Representative WBs of inflammation-related proteins interleukin-6 (IL-6) and interleukin-1β (IL-1β), p-NF-κB and the loading control (GAPDH) in WT and *Apoe* KO groups at 7 days after SCI.** (B–D)** Quantitative analysis of IL-6, IL-1β and p-NF-κB expression. Data are expressed as mean ± SEM of three independent experiments (*n* = 6 per group). *p < 0.05, WT-Sham vs. WT-SCI, IL-6, *p* = 0.0459, IL-1β, *p* = 0.0465, p-NF-κB, *p* = 0.0223; ^&&&^*p* < 0.001, KO-Sham vs. KO-SCI; IL-6, *p* < 0.001, IL-1β, *p* = 0.0002, p-NF-κB, *p* < 0.001; ^#^*p* < 0.05, ^##^*p* < 0.01, WT-SCI vs. KO-SCI, IL-6, *p* = 0.0215, IL-1β, *p* = 0.0086, p-NF-κB, *p* = 0.0162, one-way ANOVA followed by Tukey’s *post hoc* test.

Inflammatory cytokines, including IL-6 and IL-1β, aggravate cell death and inflammation after SCI (Keane et al., 2006; Oyinbo, 2011). We thus explored whether *Apoe* deficiency altered expression of these cytokines after SCI. As shown in Figure 4A, when compared with sham mice, the expression of lL-6 and IL-1β were increased in both WT and *Apoe* KO mice at 7 dpi (WT-Sham vs. WT-SCI, IL-6: 0.11 ± 0.05 vs. 0.38 ± 0.07, *p* < 0.05, IL-1β: 0.26 ± 0.02 vs. 0.93 ± 011, *p* < 0.05; KO-Sham vs. KO-SCI, IL-1β: 0.60 ± 0.09 vs. 1.77 ± 0.31, *p* < 0.001; IL-6: 0.13 ± 0.03 vs. 0.69 ± 0.11, *p* < 0.001, Figures 4A–C). Strikingly, the expression of these pro-inflammatory cytokines was higher in *Apoe* KO than in WT mice (WT-SCI vs. KO-SCI, IL-6: 0.38 ± 0.07 vs. 0.69 ± 0.11, *p* < 0.05, IL-1β: 0.93 ± 0.12 vs. 1.78 ± 0.31, *p* < 0.01, Figures 4A–C). Because activation of NF-κB promotes overproduction of inflammatory cytokines and further causes neuronal death after SCI, we examined the levels of p-NF-κB were higher in spinal cords of *Apoe* KO than WT mice at 7 dpi (WT-SCI vs. KO-SCI, 0.50 ± 0.08 vs. 0.82 ± 0.06, *p* < 0.05, Figures 4A,D). The other two representative WBs for IL-6, IL-1β and p-NF-κB are attached on Supplementary Figure S4. Taken together, these results support the hypothesis that *Apoe* deficiency induces larger production of inflammatory cytokines through activation of NF-κB following SCI.

### *Apoe* Deficiency Increases Oxidative Stress After SCI

**Figure 5:**
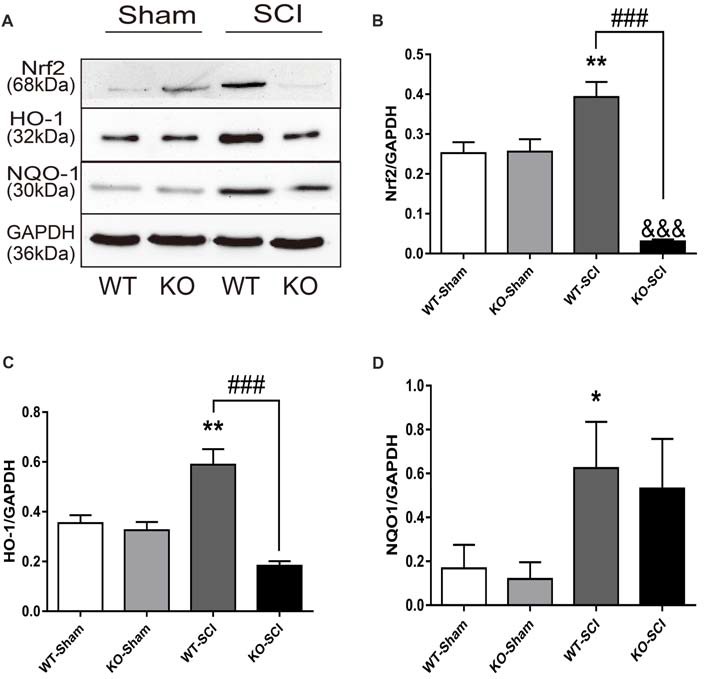
*Apoe* deficiency increases oxidative stress after SCI.** (A)** Representative WBs of antioxidative proteins Nrf2, HO-1, NQO1 and the loading control (GAPDH) in WT and *Apoe* KO groups at 7 days after SCI. **(B–D)** Quantitative analysis of Nrf2, HO-1 and NQO1 expression. Data are expressed as mean ± SEM of three independent experiments (*n* = 6 per group). *p < 0.05, **p < 0.01, WT-Sham vs. WT-SCI, Nrf2: *p* = 0.054, HO-1: *p* = 0.0013, NQO1: *p* = 0.0486; ^&&&^*p* < 0.001, KO-Sham vs. KO-SCI, Nrf2: *p* < 0.0001, HO-1: *p* = 0.0768, NQO1: *p* = 0.6424; ^###^*p* < 0.001, WT-SCI vs. KO-SCI, Nrf2, *p* < 0.0001, HO-1, *p* < 0.0001, NQO1: *p* > 0.9999; one-way ANOVA followed by Tukey’s *post hoc* test. NQO1, Kruskal-Wallis test by Dunn’s *post hoc* test.

Oxidative stress plays a crucial role in defining neuronal cell damage after SCI (Visavadiya et al., 2016). Nrf2 is a transcription factor with a strong anti-oxidative activity and anti-inflammation effects that prevents neurons the death (Campolo et al., 2017). We examined the expression of Nrf2 and its downstream signaling protein HO-1 and NQO1 by WB in mice with SCI. As is shown in Figure 5A, there were no between-group differences in either sham group. In contrast, the ablation of *Apoe* was obviously associated with a decline in Nrf2 and HO-1 expression (WT-SCI vs. KO-SCI, Nrf2: 0.39 ± 0.04 vs. 0.03 ± 0.01, *p* < 0.001; HO-1: 0.59 ± 0.06 vs. 0.18 ± 0.02, *p* < 0.001, Figures 5A–C), although the expressed of NQO1 was not significant in *Apoe* KO and WT mice at 7 dpi. There are other two representative WBs for Nrf2, HO-1 and NQO-1 presented on Supplementary Figure S5. Based on these results, the deficiency of *Apoe* appears to decrease anti-oxidative response after SCI.

### *Apoe* Deficiency Aggravates Apoptosis After SCI

**Figure 6:**
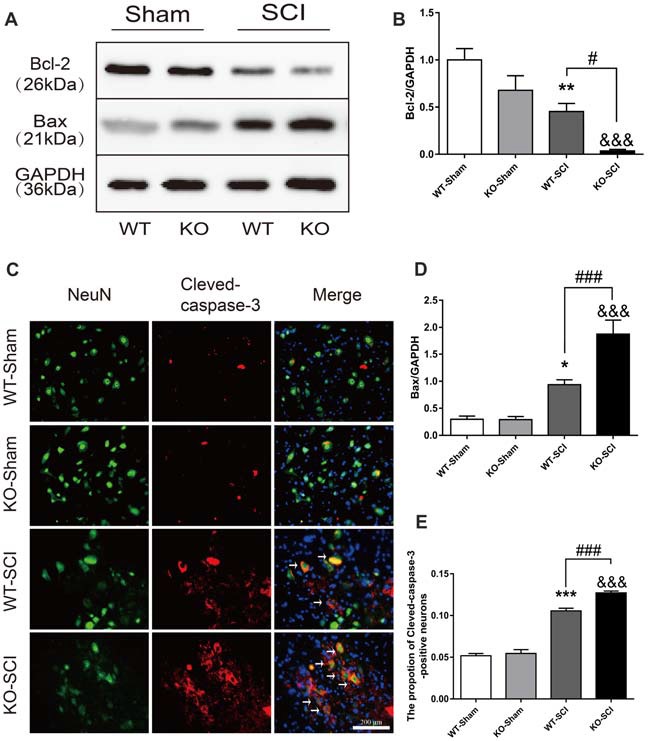
*Apoe* deficiency exacerbates neuronal apoptosis after SCI.** (A)** Representative WBs for Bcl-2 and Bax expressions and the loading control (GAPDH) in *Apoe* KO and WT group at 7 days post-injury.** (B,D)** Quantification of the expression levels of Bcl-2 and Bax. Data are expressed as mean ± SEM of three independent experiments (*n* = 6 per group). *p < 0.05, **p < 0.01, WT-Sham vs. WT-SCI, Bcl-2: *p* = 0.005, Bax: *p* = 0.0204, ^&&&^*p* < 0.001, KO-Sham vs. KO-SCI, Bcl-2: *p* = 0.0008, Bax: *p* < 0.001, ^#^*p* < 0.05, ^###^*p* < 0.001, WT-SCI vs. KO-SCI, Bcl-2: *p* = 0.0436, Bax: *p* = 0.0005, one-way ANOVA followed by Tukey’s *post hoc* test. **(C)** Representative double immunofluorescent staining for Cleaved-caspase-3-positive cells (red), NeuN (green), and DAPI (blue) in injured spinal cord of *Apoe* KO and WT mice at 7 days post-injury. **(E)** Quantification of Cleaved-caspase-3-positive cells co-labeled with NeuN to reveal apoptotic neurons. Four transverse sections were chosen at ±500 μm, ±800 μm rostral to caudal to the epicenter were obtained from each animal. Data are expressed as mean ± SEM of three independent experiments (*n* = 6 per group). ***p < 0.001, WT-Sham vs. WT-SCI, *p* < 0.0001, ^&&&^*p* < 0.001, KO-Sham vs. KO-SCI, *p* < 0.0001, ^###^*p* < 0.001, WT-SCI vs. KO-SCI, *p* = 0.0003, one-way ANOVA followed by Tukey’s *post hoc* test. Scale bar = 100 μm.

The overproduction of inflammatory mediators regulates neuronal survival, cell apoptosis, and locomotor functions after SCI (Yu and Fehlings, 2011; Cheng et al., 2016). Apoptosis occurs following SCI as a result of down-regulation of anti-apoptotic Bcl-2 and up-regulation of pro-apoptotic Bax, as well as activation of caspase-3 (Emery et al., 1998; Sastry and Rao, 2001). We observed that the levels of Bcl-2 did not differ in the spinal cord of two sham groups but was much lower in *Apoe* KO than in WT mice at 7 dpi (WT-SCI vs. KO-SCI, 0.45 ± 0.09 vs. 0.04 ± 0.02, *p* < 0.05, Figures 6A,B). Conversely, mice having *Apoe* deficiency has significantly enhanced Bax expression compared with the WT group at 7 dpi, while expression of Bax was weak in the two sham genotype groups (WT-SCI vs. KO-SCI, 0.94 ± 0.09 vs. 1.87 ± 0.26, *p* < 0.001, Figures 6A,D). To further assess the effects of APOE on neuronal apoptosis, double immunofluorescent staining was performed for Cleaved-caspase-3 and NeuN (Figure 6C). Compared with the WT group, stronger immunoreactivity of Cleaved-caspase 3-positive neurons was observed in the anterior horn of the injured spinal cord in *Apoe* KO group 7 days after injury (WT-SCI vs. KO-SCI, 0.11 ± 0.00 vs. 0.13 ± 0.00, *p* < 0.001, Figure 6E). Additional two representative WBs of Bcl2 and Bax are provided on Supplementary Figure S6. Thus, these results suggest that *Apoe* deficiency augments SCI-induced neuronal apoptosis.

### *Apoe* Deficiency Aggravates Inflammationory Response by Activating of NF-κB and Inactivating of Nrf2 After SCI

**Figure 7:**
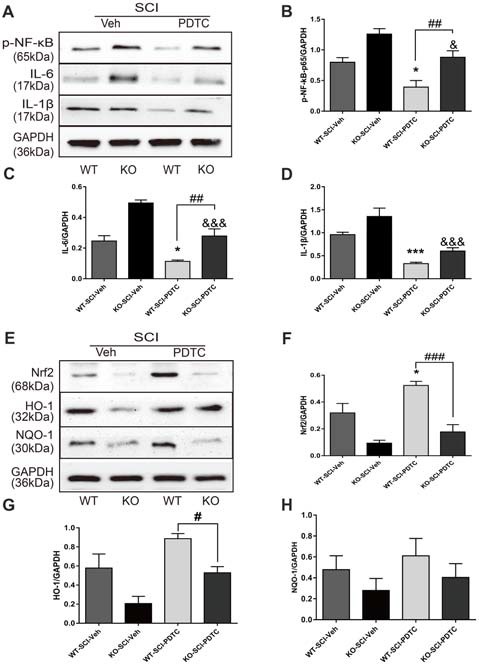
*Apoe* deficiency exacerbates inflammation by activating of NF-κB and inactivating of Nrf2 signaling pathways following SCI. **(A)** Representative immunoblots for p-NF–κB, IL-6, IL-1β and the loading control (GAPDH) in WT and *Apoe* KO groups at 7 days after SCI. **(B–D)** Quantitative levels of p-NF-κB, IL-6 and IL-1β in lesion area of *Apoe* KO and WT mice at 7 days post-injury. Data are expressed as mean ± SEM of three independent experiments (*n* = 6 per group). *p < 0.05, ***p < 0.001, WT-SCI-Veh vs. WT-SCI-PDTC, p-NF-κB: *p* = 0.0273, IL-6: *p* = 0.0193, IL-1β, *p* = 0.0005; ^&^*p* < 0.05, ^&&&^*p* < 0.001, KO-SCI-Veh vs. KO-SCI-PDTC, p-NF-κB: *p* = 0.0425, IL-6: *p* < 0.001, IL-1β: *p* < 0.0001; ^##^*p* < 0.01, ^###^*p* < 0.001, WT-SCI-PDTC vs. KO-SCI-PDTC, p-NF-κB: *p* = 0.0063, IL-6: *p* = 0.0022, IL-1β: *p* = 0.2549, one-way ANOVA followed by Tukey’s *post hoc* test. **(E)** Representative immunoblots for Nrf2, HO-1, NQO1 and the loading control (GAPDH) in WT and *Apoe* KO groups at 7 days after SCI. **(F–H)** Quantitative levels of Nrf2, HO-1, NQO1 in lesion area of *Apoe* KO and WT mice at 7 days post-injury. Data are expressed as mean ± SEM of three independent experiments (*n* = 6 per group). *p < 0.05, ***p < 0.001, WT-SCI-Veh vs. WT-SCI-PDTC, Nrf2: *p* = 0.0238, HO-1: *p* = 0.0234, NQO1: *p* > 0.05; ^&^*p* < 0.05, KO-SCI-Veh vs. KO-SCI-PDTC, Nrf2: *p* = 0.6152, HO-1: *p* = 0.0455, NQO1: *p* > 0.05; ^#^*p* < 0.05, ^###^*p* < 0.001, WT-SCI-PDTC vs. KO-SCI-PDTC, Nrf2: *p* < 0.001, HO-1: *p* = 0.0406, NQO1: *p* > 0.05, one-way ANOVA followed by Tukey’s *post hoc* test. WT-SCI and vehicle-treated group (WT-SCI-Veh), KO-SCI and vehicle-treated group (KO-SCI-Veh), WT-SCI and PDTC-treated (WT-SCI-PDTC), KO-SCI and PDTC-treated group (KO-SCI-PDTC).

To further investigate whether *Apoe* deficiency worsen secondary injury after SCI was dependent on the activation of NF-κB. PDTC, an inhibitor of the NF-κB signaling pathway, was used to block activator of NF-κB (Jiménez-Garza et al., 2005; Bell et al., 2014). WB analysis revealed that p-NF-κB protein level in both WT and KO groups with PDTC treatment was lower than in both the WT and KO groups with vehicle treatment (WT-SCI-Veh vs. WT-SCI-PDTC, 0.79 ± 0.08 vs. 0.39 ± 0.11, *p* < 0.05; KO-SCI-Veh vs. KO-SCI-PDTC, 1.26 ± 0.09 vs. 0.88 ± 0.1, *p* < 0.05, Figures 7A,B). In addition, the NF-κB protein level in WT-SCI-PDTC group was less than it in KO-SCI-PDTC (WT-SCI-PDTC vs. KO-SCI-PDTC, 0.39 ± 0.11 vs. 0.88 ± 0.11, *p* < 0.01 Figures 7A,B). Also, the expression of IL-6 was less in WT-SCI-PDTC than KO-SCI-PDTC, although the expression of IL-1β was not significance in both WT-SCI-PDTC and KO-SCI-PDTC (WT-SCI-PDTC vs. KO-SCI-PDTC, IL-6: 0.11 ± 0.01 vs. 0.28 ± 0.05, *p* < 0.01, IL-1β: 0.32 ± 0.03 vs. 0.60 ± 0.07, *p* > 0.05, Figures 7A,C,D). Because activation of Nrf2/ARE pathway also down-regulates NF-κB regulating inflammation (Mao et al., 2012; Wang et al., 2012), we analyzed whether *Apoe* deficiency increased inflammation by directly activation of NF-κB or indirectly via inactivation Nrf2-HO-1 pathway. Administration of PDTC treatment after SCI, the expression of Nrf2 and HO-1 in WT mice more increased than KO mice, but the expression of NQO-1 was no difference (WT-SCI-PDTC vs. KO-SCI-PDTC, Nrf2: 0.52 ± 0.03 vs. 0.18 ± 0.05, *p* < 0.001, HO-1: 0.89 ± 0.05 vs. 0.53 ± 0.07, *p* < 0.05; NQO-1: 0.61 ± 0.17 vs. 0.40 ± 0.13, *p* > 0.05, Figures 7E,H). The other twice representative WBs for related proteins are shown in Supplementary Figure S7. These data suggesting activation of NF-κB was indispensable for *Apoe* deficiency to enhance the release of pro-inflammation cytokines after SCI.

### *Apoe* Deficiency Promotes Inflammation and Apoptosis Independent of MAPK Signaling Pathways After SCI

To reveal the potential mechanisms through which APOE regulates SCI-induced inflammation and apoptosis in an extracellular dependent manner, we examined the activation of MAPK pathways (ERK, p-38MAPK, JNK), which are documented to regulate neuronal proliferation, inflammation, and apoptosis (Esposito et al., 2009). Interestingly, compared with sham group, p-EKR, p-p38 and p-JNK level were more elevated in WT mice at 7 days following injury (WT-Sham vs. WT-SCI, p-ERK: 0.59 ± 0.13 vs. 1.42 ± 0.19, *p* < 0.01; p-p38: 0.40 ± 0.14 vs. 0.77 ± 0.08, *p* < 0.01; p-JNK: 0.59 ± 0.07 vs. 1.19 ± 0.12, *p* < 0.05, Supplementary Figure S2). While the levels of p-JNK and p-p38 demonstrated not statistically significant in *Apoe* KO and WT mice at 7 days, *Apoe* KO mice displayed a tendency of increased levels compared with WT mice at 7 dpi (Supplementary Figure S8). Overall, these results demonstrate that MAPK pathways appear not to mediate the increased expression of inflammatory cytokines and apoptosis in *Apoe* KO mice after SCI.

## Discussion

In the present study, based on analyses of *Apoe* KO and WT mice with contused SCI, we demonstrate that *Apoe* deficiency exaggerates inflammation by increasing expression of NF-κB and inflammatory cytokines such as IL-6, IL-1β. In addition, *Apoe* deficiency reduces Nrf2-HO-1 pathway by activation of NF-κB signaling pathways. Furthermore, the depletion of *Apoe* increases neuronal apoptosis along with worsening functional outcome and increased lesion size. Here, we provide clear evidence that the mechanism of ablation of *Apoe* activated NF-κB and inactivated Nrf2-HO-1 pathway, which aggravates post-injury inflammation and oxidative stress. Our findings are important for traumatic SCI, in which *Apoe* deficiency triggers a more severe secondary cascade in the pathological process.

It is well-known that SCI is a destructive condition that irreversibly aggravates the primary injury, subsequently exacerbates the secondary damage, and leads to permanent neurological deficits (Zhao et al., 2017). This pathophysiology of secondary damage includes a series of biochemical events, including inflammation, oxidative stress, apoptosis and astroglial scar formation (Silva et al., 2014; Ahuja et al., 2017). The concept of early secondary injury mechanism is divided into immediate (<2 h), early acute (<48 h) and sub-acute (<2 weeks) stage (Rowland et al., 2008). Although there is no cure for initial traumatic injury directly damaged to spinal cord tissues, effective restraint of secondary injury plays a key role in reducing disability (Sun et al., 2016). Therefore, early therapies directed against multiple secondary damage mechanisms can be more effective to promote functional recovery after SCI. The function of APOE has drawn increasing attention, because its involvement in anti-inflammatory responses, anti-oxidative stress, and anti-apoptotic effects (Laskowitz et al., 1997; Kitagawa et al., 2002; Jiang et al., 2015), we hypothesized that its functions may block or reduce the secondary damage and improve functional recovery by NF-κB activation after SCI.

APOE plays a crucial role in modulating neuronal repair, remodeling, and protection after CNS trauma and disease (Yin et al., 2012; Saul and Wirths, 2017; Teng et al., 2017). During nerve regeneration, APOE is involved in the mobilization and reutilization of myelin-derived cholesterol for axon repair (Mahley, 1988; Li et al., 2010). Strikingly, the APOE is overexpressed in the spinal cord after SCI (Resnick et al., 2004) and serum after chronic constriction injury (Bellei et al., 2017). We found that after acute SCI in mice, APOE levels first decreased in the first 3 days, but then increased to a peak expression at 7 dpi (Figure 1A). It is well-acknowledged that activated macrophages and glia released pro-inflammatory cytokines were elevated between 30 min and 48 h following early acute SCI (Donnelly and Popovich, 2008). Several lines of evidence suggest pro-inflammatory responses decrease APOE synthesis in macrophages and glia (Gafencu et al., 2007; Yang et al., 2016). We found the APOE production gradually increased and reached the highest levels at 7 dpi. One possible function of the increased APOE production could probably suppress the inflammation in the pathological process of acute sub-stages of SCI. Endogenous APOE and exogenous APOE mimetic peptides reduced glial activation and inflammatory cytokines release after CNS disease. Interestingly, blocking inflammatory signaling effectively increases APOE expression in neuroinflammatory processes (Pocivavsek and Rebeck, 2009; Mailleux et al., 2017). These evidences suggest that APOE production and inflammation are in a negative feedback loop, with APOE suppressing inflammation and inflammation suppressing APOE production. Therefore, APOE affects changes in the inflammatory response in the pathogenesis of SCI.

Similar to findings that *Apoe* KO limits functional recovery and enlarges tissue damage in various types of nerve trauma (Herz et al., 2014; Pang et al., 2016), we and Cheng et al. (2018) have demonstrated that *Apoe* deficiency leads to more worsening anatomical and functional deficits in a mouse model of SCI. Moreover, accumulating studies have demonstrated that APOE mimic peptide could reduce apoptosis and neuroinflammation in many neurological diseases (Laskowitz et al., 2006; Wei et al., 2013; Wu et al., 2016). In addition, *APOE4*, is well known as the strongest genetic risk factor of Alzheimer’s disease (Shi et al., 2017; Underwood, 2017). Also, *APOE4* is linked with a worse neurological outcome and longer rehabilitation in patients with SCI (Jha et al., 2008). We had found *APOE4* mice have poorer locomotion performance than WT mice after SCI (Data were unshown). Notably, we found changes in the profile of endogenous APOE protein are closely related to the impaired neurological function following SCI. The behavioral function of *Apoe* KO mice was significantly poorer than that of WT mice when the APOE reached its high levels at 7 dpi (Figure 3A). Consistent with the findings that functional recovery after SCI correlates with the lesion area in the injury epicenter (Basso et al., 1996), we also observed that *Apoe* KO mice harbored larger lesion area and lower number of motor neurons than WT mice after SCI (Figures 3B–E). These histological outcomes were consistent with the functional deterioration, which indicates that APOE is important in neurologic recovery after SCI.

Although complex mechanisms mediate the severity of secondary injury after SCI, several studies have shown that overproduction of inflammatory cytokines, including lL-6 and IL-1β secreted by activated microglia and macrophages is harmful to the injured spinal cord, because they induce neuronal cell death and hinder recovery of motor function (Anwar et al., 2016; Rust and Kaiser, 2017). It is well-known that inhibiting NF-κB activation could regulate locomotive recovery through reduced lesion volume and alteration of the inflammatory environment (La Rosa et al., 2004; Brambilla et al., 2005; Bracchi-Ricard et al., 2013). We show that the absence of *Apoe* significantly increased the release of inflammatory cytokines (IL-6 and IL-1β) and activator of NF-κB after SCI. Previous studies have also shown the *Apoe*-deficiency and *APOE4* up-regulated greater proinflammatory cytokines after stimulation with LPS (Lynch et al., 2001, 2003; Ali et al., 2005). Furthermore, several evidences reported that *APOE4* affected NF-κB-mediated gene transcription that triggered a significant elevation of inflammation cytokines (Ophir et al., 2005; Shih et al., 2015; Theendakara et al., 2016). To analyze the role of the NF-κB signaling pathway in *Apoe* deficiency-induced inflammation of injured spinal cord, PDTC, an inhibitor of NF-κB nuclear translocation, was used (Bell et al., 2014). PDTC treatment resulted in a decrease of activation of NF-κB and release of inflammatory mediators in both WT-SCI-PDTC and KO-SCI-PDTC groups, indicating PDTC succeeded in suppressing activation of NF-κB. In addition*, Apoe* deficiency more increase the expression of p-NF-κB than WT mice with PDTC treatment after SCI (Figure 7). These findings support previous studies that APOE may reduce inflammation at the transcriptional level in NFκB-dependent manner. Thus, our results find that the ablation of *Apoe* increases inflammatory response by NF-κB pathway after SCI.

Also, a neuroprotective role of Nrf2, a redox-sensitive transcription factor, decrease inflammation, oxidative stress and apoptosis after SCI (Pomeshchik et al., 2014). However, studies of associations between APOE and Nrf2 in CNS have not generated clear conclusions. Pomeshchik et al. (2014) has shown that Nrf2 expression reached a peak in WT mice after SCI 7 days. And this study found APOE was significantly up-regulated and reached the highest level at the same time with Nrf2 expression. One possible reason is the increased APOE with the anti-oxidant properties may enhance the Nrf2 production. *Apoe* KO mice with diabetic gastroparesis demonstrated that decreased expression of Nrf2 elevated oxidative stress (Ravella et al., 2012). In addition, Graeser et al. (2011) have shown that Nrf2 has different regulatory effects on *Apoe* genotypes, such as *APOE4* reduces the protective role of Nrf2 in cardiovascular disease. Strikingly, as our results show in Figure 5, deficiency of *Apoe* decreased the Nrf2 signaling pathway after SCI. Furthermore, accumulating evidences shown that activation of NF-κB could directly repress Nrf2 signaling at the transcriptional level (Li et al., 2008; Yu et al., 2011). Previous researchers pointed that PDTC is a potent inducer of Nrf2 and its anti-oxidative targets in astrocytes but not neurons (Liddell et al., 2016). But the inactivation of NF-κB regulating Nrf2 pathway is elusive and rarely addressed in SCI. In the current study, PDTC-mediated NF-κB inhibition elevated Nrf2 and HO-1 level in WT mice after SCI. Of note, *Apoe*^−/−^ mice treated with PDTC administration expressed Nrf2-HO-1 still lower than WT mice after SCI. On the other hand, PDTC had no apparent effect on Nrf2 and HO-1 in *Apoe*^−/−^ mice after SCI (Figure 7). Nrf2 signaling pathways may not be directly associated with NF-κB activating inflammation induced by *Apoe* deficiency in SCI, but various signaling pathways regulate the *Apoe* is associated with Nrf2 which needs to be further investigated.

An increase in the levels of detrimental inflammatory mediators contributes to neuronal apoptosis and neurologic insult after SCI (Brambilla et al., 2005). Recent publications demonstrate that reducing neuronal apoptosis after SCI may be a favorable therapeutic strategy (Coll-Miró et al., 2016; Pei et al., 2017). It has been reported Bcl-2 protein possesses anti-apoptosis, and Bax is involved in inducing cell apoptosis (Wei et al., 2001; Bai et al., 2017). Also, Caspase-3, apoptosis-related protein markers, is activated by apoptotic stimuli and essential for apoptotic chromatin condensation and DNA (Cheng et al., 2001; Jiang et al., 2016; Xu X. et al., 2017). It is well-known that APOE was linked to neuronal apoptosis (Mahley et al., 2006; Krasemann et al., 2017). For example, APOE-containing lipoproteins binding to the low-density lipoprotein receptor-related protein prevents neuronal apoptosis (Hayashi et al., 2007). Our results show that mice lacking *Apoe* had increased apoptosis-related proteins Bax and Cleaved-caspase-3 and decreased anti-apoptotic protein Bcl-2 after SCI (Figure 6). Therefore, our conclusion is consistent with previous observations that *Apoe* deficiency up-regulates neuronal apoptosis after SCI. Yet, the exact mechanism by which *Apoe* is associated with SCI-induced neuronal apoptosis through increased inflammation needs further evaluation.

In addition, the linkage between MAPK signaling pathway and neuroprotective effects of APOE in SCI remains uncertain. A recent study shown that SCI induces activated phosphorylation of MAPK (Riego et al., 2018). Inhibition of JNK, ERK and P38 have shown that inflammation is alleviated in different animal models of pain (Ji et al., 2010). Of note, the ERK1/2 plays an important role in the activation of ApoE receptors and purified APOE3 and a tandem APOE peptide -mediated neurite outgrowth (Qiu et al., 2004). APOE receptor interaction promotes neural stem cell survival by ERK signaling, and APOE is required for oligodendrogenesis (Gan et al., 2011). However, our results of p-ERK1/2, p-p38 and p-JNK has no apparent difference in *Apoe* KO and WT mice after (Supplementary Figure S2), which seem not to support previous findings that APOE isoform-dependent inflammatory responses include NF-κB and MAPK pathways (Dose et al., 2016). A possible functional discrepancy between *Apoe* and its isoforms may explain these results. We will therefore explore the effects of *Apoe* isoforms on inflammation post-SCI in our future studies. Besides, several pieces of evidence demonstrate that APOE plays a key role in suppressing inflammation and promoting neural cell proliferation and survival by binding to its ligand (Safina et al., 2016). Because secondary damage alleviation by APOE binding to its receptor was not investigated in this study, further studies should be conducted to address this issue using appropriate techniques.

We also noted that inhibition of NF-κB leads to the histological improvement and function recovery after SCI (Church et al., 2017; He et al., 2017; Machova Urdzikova et al., 2017; Xu J. et al., 2017). For instance, selective inhibition of NF-κB signaling in astrocytes contributes to protective effects after SCI (Brambilla et al., 2005; Bracchi-Ricard et al., 2013). La Rosa et al. (2004) suggested that treatment with PDTC (30 mg/kg) significantly reduced the motor disturbance at SCI 7 days. Therefore, one limitation of the present study is that function recovery by which PDTC treatment for inhibiting the NF-κB regulated inflammation in *Apoe*^−/−^ mice after SCI, was not elaborated. Further study is crucial to elucidate this issue.

In conclusion, these findings demonstrate that: (i) *Apoe* deficiency could aggravate locomotor loss and tissue damage following SCI in mice; (ii) *Apoe* deficiency could up-regulate inflammatory cascades by NF-κB activation, and inhibiting Nrf2-HO-1 anti-oxidative pathway; and (iii) *Apoe* deficiency could exacerbate neuronal apoptosis following SCI. Although the complex mechanism by which *Apoe* knockout worsen secondary injury is not clear in this study, and at least in part through inflammatory regulation of NF-κB signaling. *Apoe*-mediated NF-κB signaling may offer a new approach to various pharmacological targets for SCI.

## Author Contributions

XY and SC wrote the main manuscript. ZS and XL performed research. SC, XL and HW collected samples. ZS and YL analyzed data. YL and CL discussed results and prepared figures. XY, CL and XM designed experiment. LM, ZZ and LB revised manuscript.

## Conflict of Interest Statement

The authors declare that the research was conducted in the absence of any commercial or financial relationships that could be construed as a potential conflict of interest.

## References

[B100] Ahuja C. S. Nori S. Tetreault L. Wilson J. Kwon B. Harrop J.. (2017). Traumatic spinal cord injury—repair and regeneration. Neurosurgery 80, S9–S22. 10.1093/neuros/nyw080 28350947

[B1] Ali K. Middleton M. Puré E. Rader D. J. (2005). Apolipoprotein E suppresses the type I inflammatory response *in vivo*. Circ. Res. 97, 922–927. 10.1161/01.res.0000187467.67684.43 16179587

[B2] Anwar M. A. Al Shehabi T. S. Eid A. H. (2016). Inflammogenesis of secondary spinal cord injury. Front. Cell. Neurosci. 10:98. 10.3389/fncel.2016.00098 27147970 PMC4829593

[B3] Bai L. Mei X. Wang Y. Yuan Y. Bi Y. Li G.. (2017). The role of netrin-1 in improving functional recovery through autophagy stimulation following spinal cord injury in rats. Front. Cell. Neurosci. 11:350. 10.3389/fncel.2017.00350 29209172 PMC5701630

[B101] Bartus K. Galino J. James N. D. Hernandez-Miranda L. R. Dawes J. M. Fricker F. R.. (2016). Neuregulin-1 controls an endogenous repair mechanism after spinal cord injury. Brain 139, 1394–1416. 10.1093/brain/aww039 26993800 PMC5477508

[B5] Basso T. Beattie M. S. Bresnahan J. C. (1996). Graded histological and locomotor outcomes after spinal cord contusion using the NYU weight-drop device versus transection. Exp. Neurol. 256, 244–256. 10.1006/exnr.1996.0098 8654527

[B4] Basso D. M. Fisher L. C. Anderson A. J. Jakeman L. Y. N. B. Tigue D. M. M. C. Popovich P. G. (2006). Basso mouse scale for locomotion detects differences in recovery after spinal cord injury in five common mouse strains. J. Neurotrauma 23, 635–659. 10.1089/neu.2006.23.635 16689667

[B6] Bell R. D. Winkler E. A. Singh I. Sagare A. P. Deane R. Wu Z.. (2014). Apolipoprotein E controls cerebrovascular integrity via cyclophilin A. Nature 21, 59–69. 10.1038/nature11087 22622580 PMC4047116

[B102] Bellei E. Vilella A. Monari E. Bergamini S. Tomasi A. Cuoghi A.. (2017). Serum protein changes in a rat model of chronic pain show a correlation between animal and humans. Sci. Rep. 7:41723. 10.1038/srep41723 28145509 PMC5286399

[B103] Bethea J. R. Castro M. Keane R. W. Lee T. T. Dietrich W. D. Yezierski R. P. (1998). Traumatic spinal cord injury induces nuclear factor-κB activation. J. Neurosci. 18, 3251–3260. 10.1523/JNEUROSCI.18-09-03251.1998 9547234 PMC6792666

[B7] Bracchi-Ricard V. Lambertsen K. L. Ricard J. Nathanson L. Karmally S. Johnstone J.. (2013). Inhibition of astroglial NF-κB enhances oligodendrogenesis following spinal cord injury. J. Neuroinflammation 10:92. 10.1186/1742-2094-10-92 23880092 PMC3751509

[B8] Brambilla R. Bracchi-Ricard V. Hu W.-H. Frydel B. Bramwell A. Karmally S.. (2005). Inhibition of astroglial nuclear factor δB reduces inflammation and improves functional recovery after spinal cord injury. J. Exp. Med. 202, 145–156. 10.1084/jem.20041918 15998793 PMC2212896

[B104] Bu G. (2010). Apolipoprotein E and its receptors in Alzheimer’s disease: pathways, pathogenesis and therapy. Nat. Rev. Neurosci. 10, 333–344. 10.1038/nrn2620 19339974 PMC2908393

[B9] Campolo M. Casili G. Biundo F. Crupi R. Cordaro M. Cuzzocrea S.. (2017). The neuroprotective effect of dimethyl fumarate in an MPTP-mouse model of Parkinson’s disease: involvement of reactive oxygen species/nuclear factor-κB/nuclear transcription factor related to NF-E2. Antioxid. Redox Signal. 27, 453–471. 10.1089/ars.2016.6800 28006954 PMC5564046

[B11] Cheng P. Kuang F. Ju G. (2016). Aescin reduces oxidative stress and provides neuroprotection in experimental traumatic spinal cord injury. Free Radic. Biol. Med. 99, 405–417. 10.1016/j.freeradbiomed.2016.09.002 27596954

[B10] Cheng E. H. A.-Y. A. Wei M. C. Weiler S. Flavell R. A. Mak T. W. Lindsten T.. (2001). BCL-2, BCL-X_L_ sequester BH3 domain-only molecules preventing BAX- and BAK-mediated mitochondrial apoptosis. Mol. Cell 8, 705–711. 10.1016/s1097-2765(01)00320-3 11583631

[B12] Cheng X. Zheng Y. Bu P. Qi X. Fan C. Li F.. (2018). Apolipoprotein E as a novel therapeutic neuroprotection target after traumatic spinal cord injury. Exp. Neurol. 299, 97–108. 10.1016/j.expneurol.2017.10.014 29056364 PMC5967384

[B13] Church J. S. Milich L. M. Lerch J. K. Popovich P. G. McTigue D. M. (2017). E6020, a synthetic TLR4 agonist, accelerates myelin debris clearance, Schwann cell infiltration, and remyelination in the rat spinal cord. Glia 65, 883–899. 10.1002/glia.23132 28251686 PMC5518478

[B14] Coll-Miró M. Francos-Quijorna I. Santos-Nogueira E. Torres-Espin A. Bufler P. Dinarello C. A.. (2016). Beneficial effects of IL-37 after spinal cord injury in mice. Proc. Natl. Acad. Sci. U S A 113, 1411–1416. 10.1073/pnas.1523212113 26787859 PMC4747716

[B15] Donnelly D. J. Popovich P. G. (2008). Inflammation and its role in neuroprotection, axonal regeneration and functional recovery after spinal cord injury. Exp. Neurol. 209, 378–388. 10.1016/j.expneurol.2007.06.009 17662717 PMC2692462

[B16] Dose J. Huebbe P. Nebel A. Rimbach G. (2016). *APOE* genotype and stress response—a mini review. Lipids Health Dis. 15:121. 10.1186/s12944-016-0288-2 27457486 PMC4960866

[B106] Elliott D. A. Kim W. S. Jans D. A. Garner B. (2007). Apoptosis induces neuronal apolipoprotein-E synthesis and localization in apoptotic bodies. Neurosci. Lett. 416, 206–210. 10.1016/j.neulet.2007.02.014 17320289

[B29] Emery E. Aldana P. Bunge M. B. Puckett W. Srinivasan A. Keane R. W.. (1998). Apoptosis after traumatic human spinal cord injury. J. Neurosurg. 89, 911–920. 10.3171/jns.1998.89.6.0911 9833815

[B17] Esposito E. Genovese T. Caminiti R. Bramanti P. Meli R. Cuzzocrea S. (2009). Melatonin reduces stress-activated/mitogen-activated protein kinases in spinal cord injury. J. Pineal Res. 46, 79–86. 10.1111/j.1600-079X.2008.00633.x 19090911

[B107] Gafencu A. V. Robciuc M. R. Fuior E. Zannis V. I. Kardassis D. Simionescu M. (2007). Inflammatory signaling pathways regulating ApoE gene expression in macrophages. J. Biol. Chem. 282, 21776–21785. 10.1074/jbc.M611422200 17553793

[B18] Gallardo G. Schlüter O. M. Südhof T. C. (2008). A molecular pathway of neurodegeneration linking α-synuclein to ApoE and Aβ peptides. Nat. Neurosci. 11, 301–308. 10.1038/nn2058 18297066

[B19] Gan H. T. Tham M. Hariharan S. Ramasamy S. Yu Y. H. Ahmed S. (2011). Identification of ApoE as an autocrine/paracrine factor that stimulates neural stem cell survival via MAPK/ERK signaling pathway. J. Neurochem. 117, 565–578. 10.1111/j.1471-4159.2011.07227.x 21352230

[B20] Graeser A. C. Boesch-Saadatmandi C. Lippmann J. Wagner A. E. Huebbe P. Storm N.. (2011). Nrf2-dependent gene expression is affected by the proatherogenic apoE4 genotype-studies in targeted gene replacement mice. J. Mol. Med. 89, 1027–1035. 10.1007/s00109-011-0771-1 21626108

[B22] Hayashi H. Campenot R. B. Vance D. E. Vance J. E. (2007). Apolipoprotein E-containing lipoproteins protect neurons from apoptosis via a signaling pathway involving low-density lipoprotein receptor-related protein-1. J. Neurosci. 27, 1933–1941. 10.1523/JNEUROSCI.5471-06.2007 17314289 PMC6673537

[B108] Hayashi H. Campenot R. B. Vance D. E. Vance J. E. (2009). Protection of neurons from apoptosis by Apolipoprotein E-containing lipoproteins does not require lipoprotein uptake and involves activation of phospholipase Cγ1 and inhibition of calcineurin. J. Biol. Chem. 284, 29605–29613. 10.1074/jbc.M109.039560 19717566 PMC2785593

[B23] He Z. Zhou Y. Lin L. Wang Q. Khor S. Mao Y.. (2017). Dl-3-n-butylphthalide attenuates acute inflammatory activation in rats with spinal cord injury by inhibiting microglial TLR4/NF-κB signalling. J. Cell. Mol. Med. 21, 3010–3022. 10.1111/jcmm.13212 28842949 PMC5661102

[B24] Herz J. Hagen S. I. Bergmüller E. Sabellek P. Göthert J. R. Buer J.. (2014). Exacerbation of ischemic brain injury in hypercholesterolemic mice is associated with pronounced changes in peripheral and cerebral immune responses. Neurobiol. Dis. 62, 456–468. 10.1016/j.nbd.2013.10.022 24184800

[B25] Hong L. T. A. Kim Y. M. Park H. H. Hwang D. H. Cui Y. Lee E. M.. (2017). An injectable hydrogel enhances tissue repair after spinal cord injury by promoting extracellular matrix remodeling. Nat. Commun. 8:533. 10.1038/s41467-017-00583-8 28912446 PMC5599609

[B109] Iwata A. Browne K. D. Chen X.-H. Yuguchi T. Smith D. H. (2005). Traumatic brain injury induces biphasic upregulation of ApoE and ApoJ protein in rats. J. Neurosci. Res. 82, 103–114. 10.1002/jnr.20607 16118797

[B26] Jha A. Lammertse D. P. Coll J. R. Charlifue S. Coughlin C. T. Whiteneck G. G.. (2008). Apolipoprotein E epsilon4 allele and outcomes of traumatic spinal cord injury. J. Spinal Cord Med. 31, 171–176. 10.1080/10790268.2008.11760708 18581664 PMC2565476

[B27] Jiang X. Li L. Ying Z. Pan C. Huang S. Li L.. (2016). A small molecule that protects the integrity of the electron transfer chain blocks the mitochondrial apoptotic pathway. Mol. Cell 63, 229–239. 10.1016/j.molcel.2016.06.016 27447985

[B110] Jiang L. Zhong J. Dou X. Cheng C. Huang Z. Sun X. (2015). Effects of ApoE on intracellular calcium levels and apoptosis of neurons after mechanical injury. Neuroscience 301, 375–383. 10.1016/j.neuroscience.2015.06.005 26073697

[B56] Ji R. R. Gereau R. W. IV. Malcangio M. Strichartz G. R. (2010). MAP kinase and pain. Brain Res. Rev. 60, 135–148. 10.1016/j.brainresrev.2008.12.011 19150373 PMC2666786

[B28] Jiménez-Garza O. Camacho J. Ibarra A. Martínez A. Guízar-Sahagún G. (2005). Early effects of modulating nuclear factor-δB activation on traumatic spinal cord injury in rats. Ann. N Y Acad. Sci. 1053, 148–150. 10.1196/annals.1344.012 16179517

[B111] Kanninen K. M. Pomeshchik Y. Leinonen H. Malm T. Koistinaho J. Levonen A. L. (2015). Applications of the Keap1-Nrf2 system for gene and cell therapy. Free Radic. Biol. Med. 88, 350–361. 10.1016/j.freeradbiomed.2015.06.037 26164630

[B30] Keane R. W. Davis A. R. Dietrich W. D. (2006). Inflammatory and apoptotic signaling after spinal cord injury. J. Neurotrauma 23, 335–344. 10.1089/neu.2006.23.335 16629620

[B112] Kim E. Woo M.-S. Qin L. Ma T. Beltran C. D. Bao Y.. (2015). Daidzein augments cholesterol homeostasis via ApoE to promote functional recovery in chronic stroke. J. Neurosci. 35, 15113–15126. 10.1523/JNEUROSCI.2890-15.2015 26558782 PMC4642242

[B113] Kitagawa K. Matsumoto M. Kuwabara K. Takasawa K. Tanaka S. Sasaki T.. (2002). Protective effect of apolipoprotein E against ischemic neuronal injury is mediated through antioxidant action. J. Neurosci. Res. 68, 226–232. 10.1002/jnr.10209 11948667

[B31] Krasemann S. Madore C. Cialic R. Baufeld C. Calcagno N. El Fatimy R.. (2017). The TREM2-APOE pathway drives the transcriptional phenotype of dysfunctional microglia in neurodegenerative diseases. Immunity 47, 566.e9–581.e9. 10.1016/j.immuni.2017.08.008 28930663 PMC5719893

[B114] Lang B. T. Cregg J. M. DePaul M. A. Tran A. P. Xu K. Dyck S. M.. (2015). Modulation of the proteoglycan receptor PTPσ promotes recovery after spinal cord injury. Nature 518, 404–408. 10.1038/nature13974 25470046 PMC4336236

[B32] La Rosa G. Cardali S. Genovese T. Conti A. Di Paola R. La Torre D.. (2004). Inhibition of the nuclear factor-κB activation with pyrrolidine dithiocarbamate attenuating inflammation and oxidative stress after experimental spinal cord trauma in rats. J. Neurosurg. 3, 311–321. 10.3171/spi.2004.1.3.0311 15478370

[B115] Laskowitz D. T. Goel S. Bennett E. R. Matthew W. D. (1997). Apolipoprotein E suppresses glial cell secretion of TNFα. J. Neuroimmunol. 76, 70–74. 10.1016/s0165-5728(97)00021-0 9184634

[B116] Laskowitz D. T. Horsburgh K. Roses A. D. (1998). Apolipoprotein E and the CNS response to injury. J. Cereb. Blood Flow Metab. 18, 465–471. 10.1097/00004647-199805000-00001 9591838

[B33] Laskowitz D. T. Fillit H. Yeung N. Toku K. Vitek M. P. (2006). Apolipoprotein E-derived peptides reduce CNS inflammation: implications for therapy of neurological disease. Acta Neurol. Scand. 114, 15–20. 10.1111/j.1600-0404.2006.00680.x 16866906

[B34] Li J. Chen S. Zhao Z. Luo Y. Hou Y. Li H. (2017). Effect of VEGF on inflammatory regulation, neural survival, and functional improvement in rats following a complete spinal cord transection. Front. Cell. Neurosci. 11:381. 10.3389/fncel.2017.00381 29238292 PMC5712574

[B118] Li X.-Z. Dinga Y.-Z. Wu H.-F. Bian Z. P. Xu J. D. Gu C. R.. (2017). Astragaloside IV prevents cardiac remodeling in the Apolipoprotein E-deficient mice by regulating cardiac homeostasis and oxidative stress. Cell. Physiol. Biochem. 44, 2422–2438. 10.1159/000486166 29268252

[B117] Li F.-Q. Fowler K. A. Neil J. E. Colton C. A. Vitek M. P. (2010). An apolipoprotein E-mimetic stimulates axonal regeneration and remyelination after peripheral nerve injury. J. Pharmacol. Exp. Ther. 334, 106–115. 10.1124/jpet.110.167882 20406857 PMC2912037

[B36] Li W. Khor T. O. Xu C. Shen G. Jeong W. Yu S.. (2008). Activation of Nrf2-antioxidant signaling attenuates NFδB-inflammatory response and elicits apoptosis §. Biochem. Pharmacol. 76, 1485–1489. 10.1016/j.bcp.2008.07.017 18694732 PMC2610259

[B35] Li L. Oppenheim R. W. Lei M. Houenou L. J. (1994). Neurotrophic agents prevent motoneuron death following sciatic nerve section in the neonatal mouse. J. Neurobiol. 25, 759–766. 10.1002/neu.480250702 8089654

[B119] Liu C.-C. Zhao N. Fu Y. Wang N. Linares C. Tsai C.-W.. (2017). ApoE4 accelerates early seeding of amyloid pathology. Neuron 96, 1024.e3–1032.e3. 10.1016/j.neuron.2017.11.013 29216449 PMC5948105

[B37] Liddell J. R. Lehtonen S. Duncan C. Keksa-Goldsteine V. Levonen A.-L. Goldsteins G.. (2016). Pyrrolidine dithiocarbamate activates the Nrf2 pathway in astrocytes. J. Neuroinflammation 13:49. 10.1186/s12974-016-0515-9 26920699 PMC4768425

[B120] Lu M. Wang S. Han X. Lv D. (2013). Butein inhibits NF-κB activation and reduces infiltration of inflammatory cells and apoptosis after spinal cord injury in rats. Neurosci. Lett. 542, 87–91. 10.1016/j.neulet.2013.03.004 23499960

[B38] Lynch J. R. Morgan D. Mance J. Matthew W. D. Laskowitz D. T. (2001). Apolipoprotein E modulates glial activation and the endogenous central nervous system inflammatory response. J. Neuroimmunol. 114, 107–113. 10.1016/s0165-5728(00)00459-8 11240021

[B39] Lynch J. R. Tang W. Wang H. Vitek M. P. Bennett E. R. Sullivan P. M.. (2003). APOE genotype and an ApoE-mimetic peptide modify the systemic and central nervous system inflammatory response. J. Biol. Chem. 278, 48529–48533. 10.1074/jbc.m306923200 14507923

[B40] Ma M. Basso D. M. Walters P. Stokes B. T. Jakeman L. B. (2001). Behavioral and histological outcomes following graded spinal cord contusion injury in the C57Bl/6 mouse. Exp. Neurol. 169, 239–254. 10.1006/exnr.2001.7679 11358439

[B41] Machova Urdzikova L. Ruzicka J. Karova K. Kloudova A. Svobodova B. Amin A.. (2017). A green tea polyphenol epigallocatechin-3-gallate enhances neuroregeneration after spinal cord injury by altering levels of inflammatory cytokines. Neuropharmacology 126, 213–223. 10.1016/j.neuropharm.2017.09.006 28899730

[B121] Mahley R. W. (1988). Apolipoprotein E: cholesterol transport protein with expanding role in cell biology. Science 240, 622–630. 10.1126/science.3283935 3283935

[B122] Mahley R. W. Huang Y. (2012). Apolipoprotein E sets the stage: response to injury triggers neuropathology. Neuron 76, 871–885. 10.1016/j.neuron.2012.11.020 23217737 PMC4891195

[B42] Mahley R. W. Weisgraber K. H. Huang Y. (2006). Apolipoprotein E4: a causative factor and therapeutic target in neuropathology, including Alzheimer’s disease. Proc. Natl. Acad. Sci. U S A 103, 5644–5651. 10.1073/pnas.0600549103 16567625 PMC1414631

[B123] Mailleux J. Timmermans S. Nelissen K. Vanmol J. Vanmierlo T. van Horssen J.. (2017). Low-density lipoprotein receptor deficiency attenuates neuroinflammation through the induction of Apolipoprotein E. Front. Immunol. 8:1701. 10.3389/fimmu.2017.01701 29276512 PMC5727422

[B124] Mao L. Wang H. Qiao L. Wang X. (2010). Disruption of Nrf2 enhances the upregulation of nuclear factor-κB activity, tumor necrosis factor- α, and matrix metalloproteinase-9 after spinal cord injury in mice. Mediators Inflamm. 2010:238321. 10.1155/2010/238321 20862369 PMC2938451

[B43] Mao L. Wang H. Wang X. Tian L. Xu J. Y. (2012). Disruption of Nrf2 exacerbated the damage after spinal cord injury in mice. J. Trauma Acute Care Surg. 72, 189–198. 10.1097/TA.0b013e31821bf541 21926641

[B44] Narang A. Qiao F. Atkinson C. Zhu H. Yang X. Kulik L.. (2017). Natural IgM antibodies that bind neoepitopes exposed as a result of spinal cord injury, drive secondary injury by activating complement. J. Neuroinflammation 14:120. 10.1186/s12974-017-0894-6 28629465 PMC5477255

[B125] Nuriel T. Peng K. Y. Ashok A. Dillman A. A. Mathews H. Y. Apuzzo J.. (2017). The endosomal—lysosomal pathway is dysregulated by APOE4 expression *in vivo*. Front. Neurosci. 11:702. 10.3389/fnins.2017.00702 29311783 PMC5733017

[B46] Ophir G. Amariglio N. Jacob-Hirsch J. Elkon R. Rechavi G. Michaelson D. M. (2005). Apolipoprotein E4 enhances brain inflammation by modulation of the NF-κB signaling cascade. Neurobiol. Dis. 20, 709–718. 10.1016/j.nbd.2005.05.002 15979312

[B47] Oyinbo C. A. (2011). Secondary injury mechanisms in traumatic spinal cord injury: a nugget of this multiply cascade. Acta Neurobiol. Exp. 71, 281–299. 21731081 10.55782/ane-2011-1848

[B48] Pang J. Wu Y. Peng J. Yang P. Kuai L. Qin X.. (2016). Potential implications of Apolipoprotein E in early brain injury after experimental subarachnoid hemorrhage: involvement in the modulation of blood-brain barrier integrity. Oncotarget 7, 56030–56044. 10.18632/oncotarget.10821 27463015 PMC5302894

[B49] Pei J. Fan L. Nan K. Li J. Dang X. Wang K. (2017). HSYA alleviates secondary neuronal death through attenuating oxidative stress, inflammatory response, and neural apoptosis in SD rat spinal cord compression injury. J. Neuroinflammation 14:97. 10.1186/s12974-017-0870-1 28468657 PMC5415746

[B126] Pocivavsek A. Rebeck G. W. (2009). Inhibition of c-Jun N-terminal kinase increases apoE expression *in vitro* and *in vivo*. Biochem. Biophys. Res. Commun. 387, 516–520. 10.1016/j.bbrc.2009.07.048 19615334 PMC2745314

[B50] Pomeshchik Y. Kidin I. Savchenko E. Rolova T. Yamamoto M. Levonen A.-L.. (2014). Does Nrf2 gene transfer facilitate recovery after contusion spinal cord injury? Antioxid. Redox Signal. 20, 1313–1323. 10.1089/ars.2013.5453 23841575

[B52] Qiu Z. Hyman B. T. Rebeck G. W. (2004). Apolipoprotein E receptors mediate neurite outgrowth through activation of p44/42 mitogen-activated protein kinase in primary neurons. J. Biol. Chem. 279, 34948–34956. 10.1074/jbc.M401055200 15169786

[B127] Rafati D. S. Geissler K. Johnson K. Unabia G. Hulsebosch C. Nesic-Taylor O.. (2008). Nuclear factor-κB decoy amelioration of spinal cord injury-induced inflammation and behavior outcomes. J. Neurosci. Res. 86, 566–580. 10.1002/jnr.21508 17918744

[B128] Ramer L. M. Ramer M. S. Bradbury E. J. (2014). Restoring function after spinal cord injury: towards clinical translation of experimental strategies. Lancet Neurol. 13, 1241–1256. 10.1016/s1474-4422(14)70144-9 25453463

[B53] Ravella K. Yang H. Gangula P. R. R. (2012). Impairment of gastric nitrergic and NRF2 system in Apolipoprotein E knockout mice. Dig. Dis. Sci. 57, 1504–1509. 10.1007/s10620-012-2070-2 22302246 PMC3677538

[B129] Resnick D. K. Schmitt C. Miranpuri G. S. Dhodda V. K. Isaacson J. Vemuganti R. (2004). Molecular evidence of repair and plasticity following spinal cord injury. Neuroreport 15, 837–839. 10.1097/00001756-200404090-00020 15073526

[B54] Riego G. Redondo A. Leánez S. Pol O. (2018). Mechanism implicated in the anti-allodynic and anti-hyperalgesic effects induced by the activation of heme oxygenase 1/carbon monoxide signaling pathway in the central nervous system of mice with neuropathic pain. Biochem. Pharmacol. 148, 52–63. 10.1016/j.bcp.2017.12.007 29247614

[B51] Rowland J. W. Hawryluk G. W. Kwon B. Fehlings M. G. (2008). Current status of acute spinal cord injury pathophysiology and emerging therapies: promise on the horizon. Neurosurg. Focus 25:E2. 10.3171/FOC.2008.25.11.E2 18980476

[B57] Rust X. R. Kaiser J. (2017). Insights into the dual role of inflammation after spinal cord injury. J. Neurosci. 37, 4658–4660. 10.1523/JNEUROSCI.0498-17.2017 28469010 PMC6596496

[B58] Safina D. Schlitt F. Romeo R. Pflanzner T. Pietrzik C. U. Narayanaswami V.. (2016). Low-density lipoprotein receptor-related protein 1 is a novel modulator of radial glia stem cell proliferation, survival, and differentiation. Glia 64, 1363–1380. 10.1002/glia.23009 27258849 PMC5033964

[B59] Sastry P. S. Rao K. S. (2001). Apoptosis and the nervous system. J. Neurochem. 74, 1–20. 10.1046/j.1471-4159.2000.0740001.x 10617101

[B130] Saul A. Wirths O. (2017). Endogenous Apolipoprotein E (ApoE) fragmentation is linked to amyloid pathology in transgenic mouse models of Alzheimer’s disease. Mol. Neurobiol. 54, 319–327. 10.1007/s12035-015-9674-4 26742512

[B131] Seitz A. Kragol M. Aglow E. Showe L. Heber-Katz E. (2003). Apolipoprotein E expression after spinal cord injury in the mouse. J. Neurosci. Res. 71, 417–426. 10.1002/jnr.10482 12526030

[B60] Shi Y. Yamada K. Liddelow S. A. Smith S. T. Zhao L. Luo W.. (2017). ApoE4 markedly exacerbates tau-mediated neurodegeneration in a mouse model of tauopathy. Nature 549, 523–527. 10.1038/nature24016 28959956 PMC5641217

[B61] Shih R.-H. Wang C.-Y. Yang C.-M. (2015). NF-κB signaling pathways in neurological inflammation: a mini review. Front. Mol. Neurosci. 8:77. 10.3389/fnmol.2015.00077 26733801 PMC4683208

[B132] Silva N. A. Sousa N. Reis R. L. Salgado A. J. (2014). From basics to clinical: a comprehensive review on spinal cord injury. Prog. Neurobiol. 114, 25–57. 10.1016/j.pneurobio.2013.11.002 24269804

[B133] Sun X. Jones Z. B. Chen X. Zhou L. So K.-F. Ren Y. (2016). Multiple organ dysfunction and systemic inflammation after spinal cord injury: a complex relationship. J. Neuroinflammation 13:260. 10.1186/s12974-016-0736-y 27716334 PMC5053065

[B134] Teng Z. Guo Z. Zhong J. Cheng C. Huang Z. Wu Y.. (2017). ApoE influences the blood-brain barrier through the NF-κB/MMP-9 pathway after traumatic brain injury. Sci. Rep. 7:6649. 10.1038/s41598-017-06932-3 28751738 PMC5532277

[B62] Theendakara V. Peters-libeu C. A. Spilman X. P. Poksay K. S. Bredesen D. E. Rao R. V. (2016). Direct transcriptional effects of Apolipoprotein E. J. Neurosci. 36, 685–700. 10.1523/JNEUROSCI.3562-15.2016 26791201 PMC4719010

[B63] Underwood E. (2017). How ApoE4 endangers brains. Science 357:1224. 10.1126/science.357.6357.1224 28935785

[B64] Visavadiya N. P. Patel S. P. VanRooyen J. L. Sullivan P. G. Rabchevsky A. G. (2016). Cellular and subcellular oxidative stress parameters following severe spinal cord injury. Redox Biol. 8, 59–67. 10.1016/j.redox.2015.12.011 26760911 PMC4712315

[B45] Wang X. de Rivero Vaccari J. P. Wang H. Diaz P. German R. Marcillo A. E.. (2012). Activation of the nuclear factor E2-related factor 2/antioxidant response element pathway is neuroprotective after spinal cord injury. J. Neurotrauma 29, 936–945. 10.1089/neu.2011.1922 21806470 PMC3303102

[B135] Wang R. Hong J. Lu M. Neil J. E. Vitek M. P. Liu X.. (2014). ApoE mimetic ameliorates motor deficit and tissue damage in rat spinal cord injury. J. Neurosci. Res. 92, 884–892. 10.1002/jnr.23371 24633884

[B136] Wang L. Yao Y. He R. Meng Y. Li N. Zhang D.. (2017). Methane ameliorates spinal cord ischemia-reperfusion injury in rats: antioxidant, anti-inflammatory and anti-apoptotic activity mediated by Nrf2 activation. Free Radic. Biol. Med. 103, 69–86. 10.1016/j.freeradbiomed.2016.12.014 28007572

[B65] Wei J. Zheng M. Liang P. Wei Y. Yin X. Tang Y.. (2013). Apolipoprotein E and its mimetic peptide suppress Th1 and Th17 responses in experimental autoimmune encephalomyelitis. Neurobiol. Dis. 56, 59–65. 10.1016/j.nbd.2013.04.009 23619428

[B66] Wei M. C. Zong W. Cheng E. H. Lindsten T. Panoutsakopoulou V. Ross A. J.. (2001). Proapoptotic BAX and BAK: a requisite gateway to mitochondrial dysfunction and death. Science 292, 727–730. 10.1126/science.1059108 11326099 PMC3049805

[B67] Wu Y. Pang J. Peng J. Cao F. Vitek M. P. Li F.. (2016). An ApoE-derived mimic peptide, COG1410, alleviates early brain injury via reducing apoptosis and neuroinflammation in a mouse model of subarachnoid hemorrhage. Neurosci. Lett. 627, 92–99. 10.1016/j.neulet.2016.05.058 27241720

[B68] Xu J. He J. He H. Peng R. Xi J. (2017). TWEAK-Fn14 influences neurogenesis status via modulating NF-κB in mice with spinal cord injury. Mol. Neurobiol. 54, 7497–7506. 10.1007/s12035-016-0248-x 27822714

[B69] Xu X. Huang E. Tai Y. Zhao X. Chen X. Chen C.. (2017). Nupr1 modulates dopaminergic neuronal apoptosis and autophagy through CHOP-Trib3-mediated endoplasmic reticulum stress signaling pathway. Front. Mol. Neurosci. 10:203. 10.3389/fnmol.2017.00203 28694771 PMC5483452

[B137] Yang L. Liu C.-C. Zheng H. Kanekiyo T. Atagi Y. Jia L.. (2016). LRP1 modulates the microglial immune response via regulation of JNK and NF-κB signaling pathways. J. Neuroinflammation 13:304. 10.1186/s12974-016-0772-7 27931217 PMC5146875

[B138] Yin C. Zhou S. Jiang L. Sun X. (2012). Mechanical injured neurons stimulate astrocytes to express apolipoprotein E through ERK pathway. Neurosci. Lett. 515, 77–81. 10.1016/j.neulet.2012.03.023 22450050

[B55] Yu W. R. Fehlings M. G. (2011). Fas/FasL-mediated apoptosis and inflammation are key features of acute human spinal cord injury: implications for translational, clinical application. Acta Neuropathol. 122, 747–761. 10.1007/s00401-011-0882-3 22038545 PMC3224722

[B70] Yu M. Li H. Liu Q. Liu F. Tang L. Li C.. (2011). Nuclear factor p65 interacts with Keap1 to repress the Nrf2-ARE pathway. Cell. Signal. 23, 883–892. 10.1016/j.cellsig.2011.01.014 21262351

[B139] Zhao H. Chen S. Gao K. Zhou Z. Wang C. Shen Z.. (2017). Resveratrol protects against spinal cord injury by activating autophagy and inhibiting apoptosis mediated by the SIRT1/AMPK signaling pathway. Neuroscience 348, 241–251. 10.1016/j.neuroscience.2017.02.027 28238848

[B140] Zheng H. Jia L. Liu C.-C. Rong Z. Zhong L. Yang L.. (2017). TREM2 promotes microglial survival by activating Wnt/β-catenin pathway. J. Neurosci. 37, 1772–1784. 10.1523/JNEUROSCI.2459-16.2017 28077724 PMC5320608

