# Supplementary material for: Apolipoprotein E Deficiency Exacerbates Spinal Cord Injury in Mice: Inflammatory Response and Oxidative Stress Mediated by NF-κB Signaling Pathway
Source: Front Cell Neurosci. 2018 May 23;12:142. doi: 10.3389/fncel.2018.00142 (PMC5974465; doi:10.3389/fncel.2018.00142)
Supplement: Supplementary file 1 [file Data_Sheet_1.pdf]

## *Supplementary Material*

# Apolipoprotein E Deficiency Exacerbates Spinal Cord Injury in Mice: Inflammatory Response and Oxidative Stress Mediated by NF-κB Signaling Pathway

***Xuan Yang[1†], Shurui Chen[2†], Zhenya Shao[2], Yuanlong Li[2], He Wu[3], Xian Li[4], Liang Mao[5], Zipeng Zhou[2], Liangjie Bai[6], Xifan Mei[2]* and Chang Liu[3]****

*** Correspondence:** *Xifan Mei, Email: meixifan1971@163.com*

*Chang Liu, Email: liuchang1971mei@163.com*

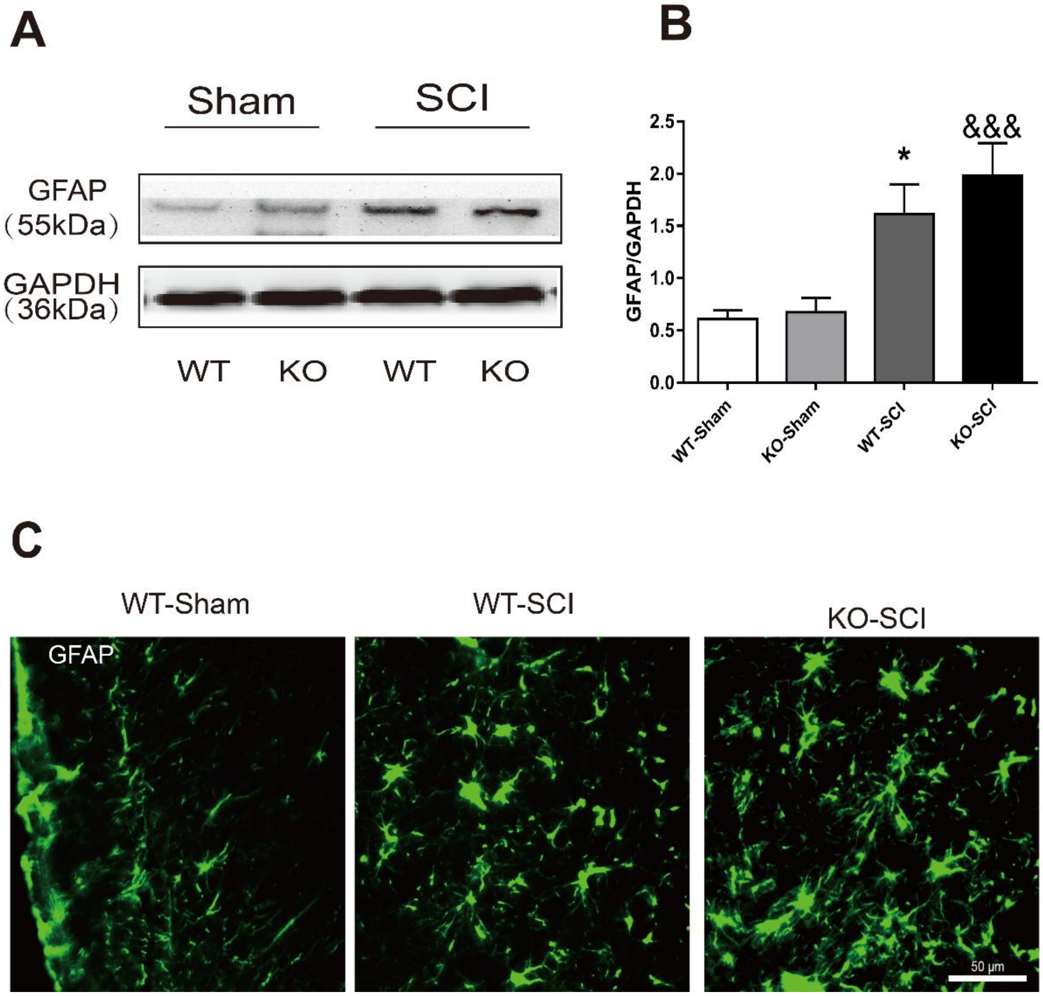


**Supplementary Figure 1**. **Effects of *Apoe* deficiency on reactive astrogliosis after SCI.**

**(A)** Representative Western blots of GFAP and the loading control (GAPDH) in WT and *Apoe* KO groups at 7 days after SCI.

**(B)** Quantitative analysis of GFAP expression in WT and *Apoe* KO groups at 7 days after SCI. Data are expressed as mean ± SEM of 3 independent experiments, (n = 6 per group). **p* < 0.05, WT-Sham vs. WT-SCI, GFAP, *p* = 0.0189, &&&*p* < 0.001, KO-Sham vs. KO-SCI, *p* = 0.0018, one-way ANOVA followed by Tukey's post hoc test.

**(C)** Representative images of GFAP staining in WT and *Apoe* KO groups at 7 days after SCI. Four transverse sections were chosen at ±1000 μm rostral to caudal to the epicenter were obtained from each animal. Scale bar = 50 μm.

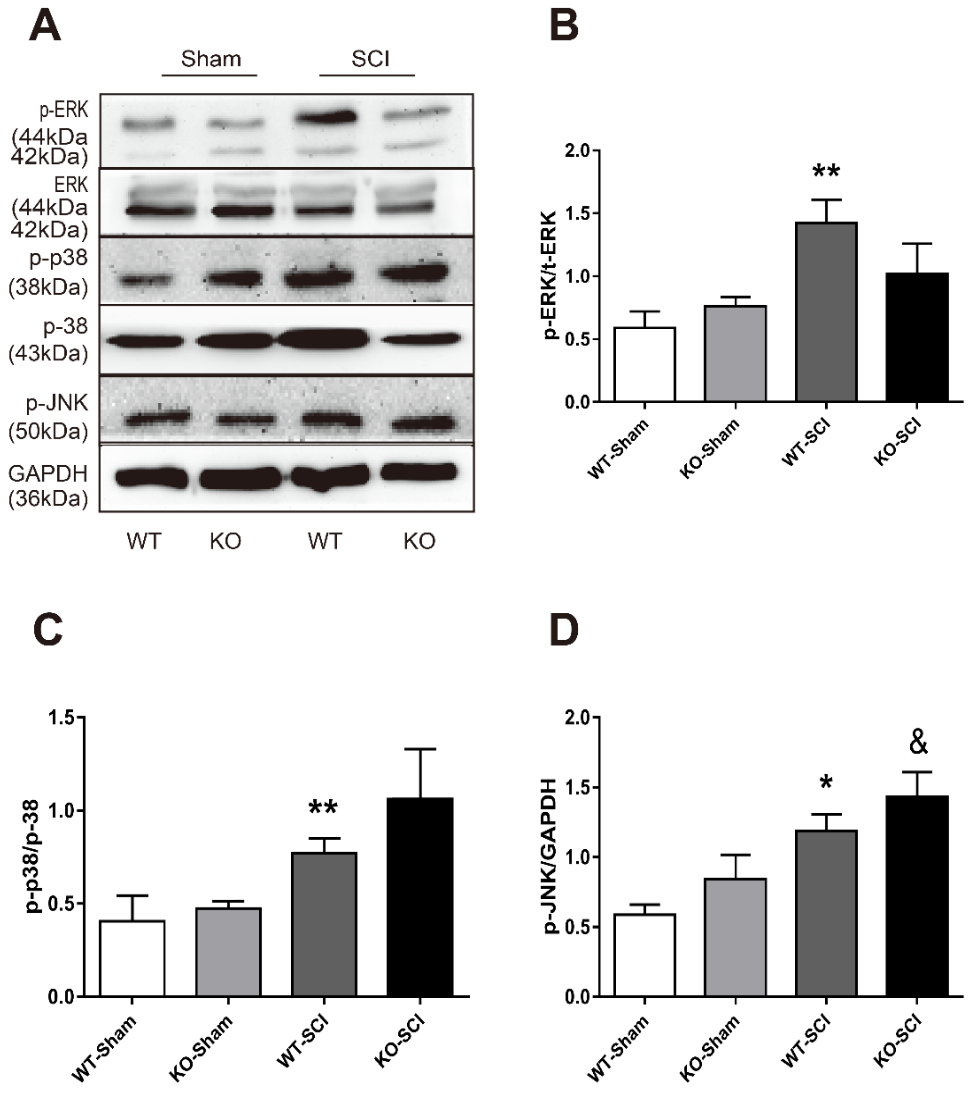


**Supplementary Figure 2. Effects of *Apoe* deficiency are independent of MAPK signaling pathway after SCI.**

**(A)** Representative immunoblots for p-ERK, p-p38, p-JNK and the loading control (GAPDH) in WT and APOE KO mice at 7 days after SCI.

**(B, C, D)** Quantification of MAPK pathway proteins. Data are expressed as mean ± SEM of 3 independent experiments, (n= 6 per group). **p* < 0.05, ***p* < 0.01, WT-Sham vs. WT-SCI,

p-ERK/T-ERK, $p = 0.0062$, p-p38/p-38, $p = 0.0096$, p-JNK, $p = 0.0253$; &p < 0.05, KO-Sham vs. KO-SCI, p-JNK: $p = 0.0288$, one-way ANOVA followed by Tukey's post hoc test. P-p38/p-38, Kruskal-Wallis test by Dunn's post hoc test.

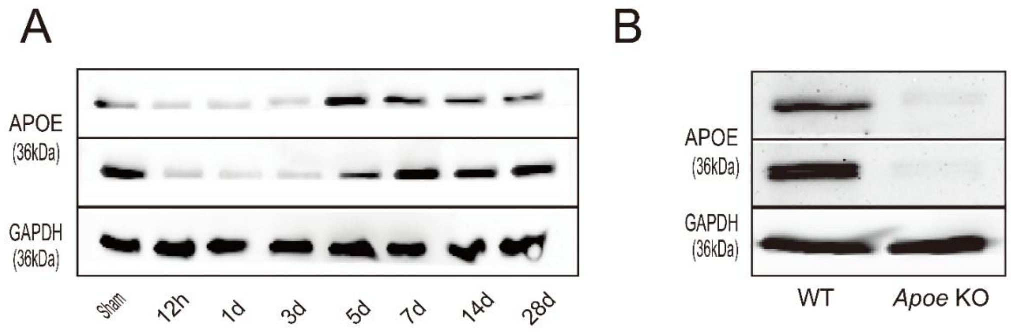


**Supplementary Figure 3**. **Representative western blots for APOE protein assessed in the spinal cord.**

**(A)** APOE expression at the specified time points after SCI by WB. (n = 6/group).
**(B)** APOE expression was identified in spinal cord in WT and *Apoe* KO mice by WB (n = 6/group).

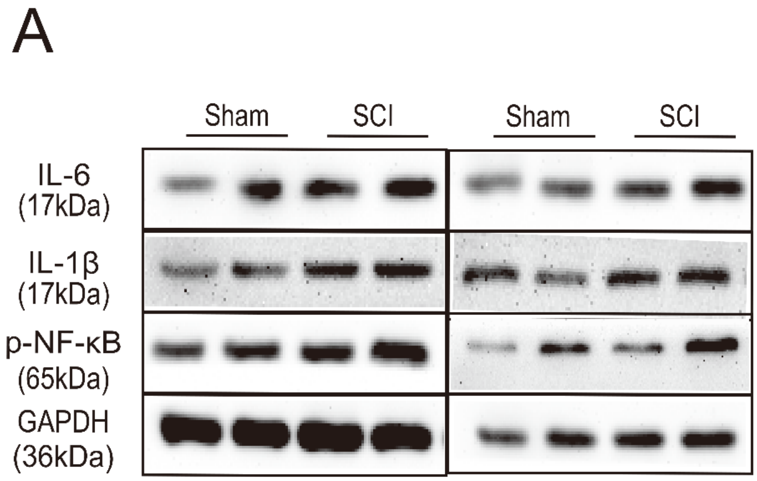


**Supplementary Figure 4: *Apoe* KO mice increased proinflammatory cytokines expression compared with WT mice after SCI.**

**(A)** Representative Western blots for IL-6, IL-1β and p-NF-κB expression in spinal cord *Apoe* KO mice after 7 days SCI. (n = 6/group).

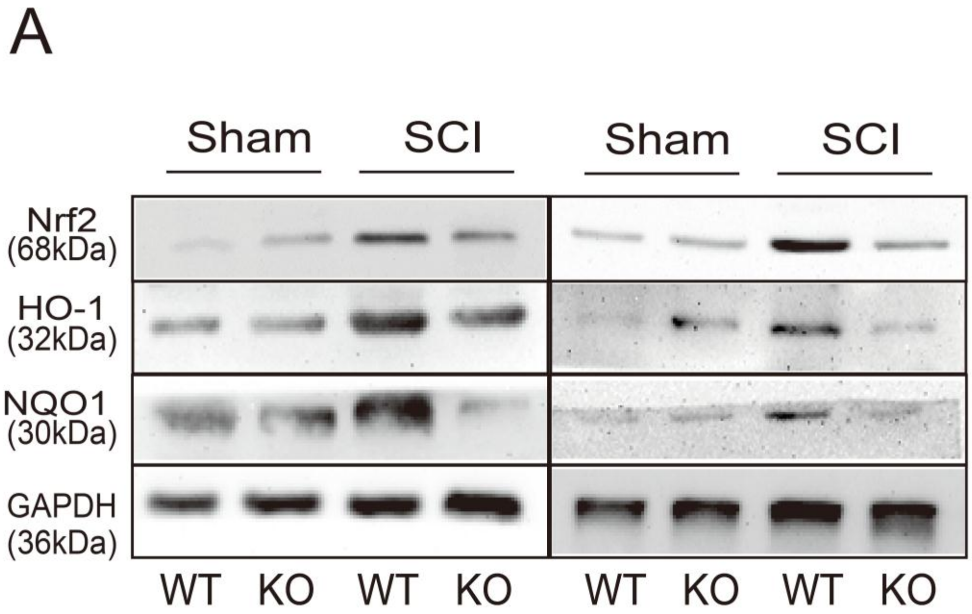


**Supplementary Figure 5: *Apoe* KO mice decreased Nrf2-HO-1 pathway compared with WT mice after SCI.**

**(A)** Representative Western blots for Nrf2, HO-1 and NQO1 expression in WT and *Apoe* KO mice after 7 days SCI. (n = 6/group).

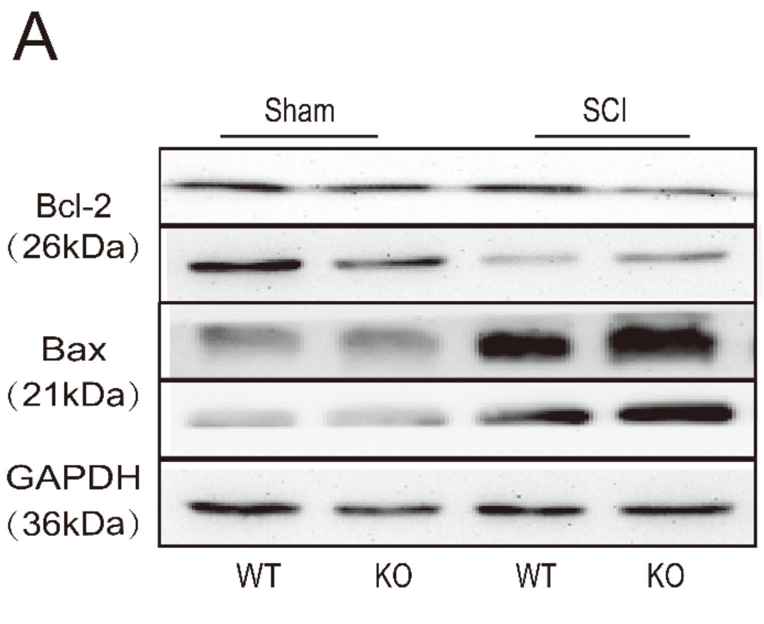


**Supplementary Figure 6: *Apoe* KO mice shows more apoptosis after SCI compared to the WT mice.**

**(A)** WB was performed to assess Bcl-2 and Bax expression in spinal cord in WT and *Apoe* KO mice at 7 days after SCI. (n = 6/group).

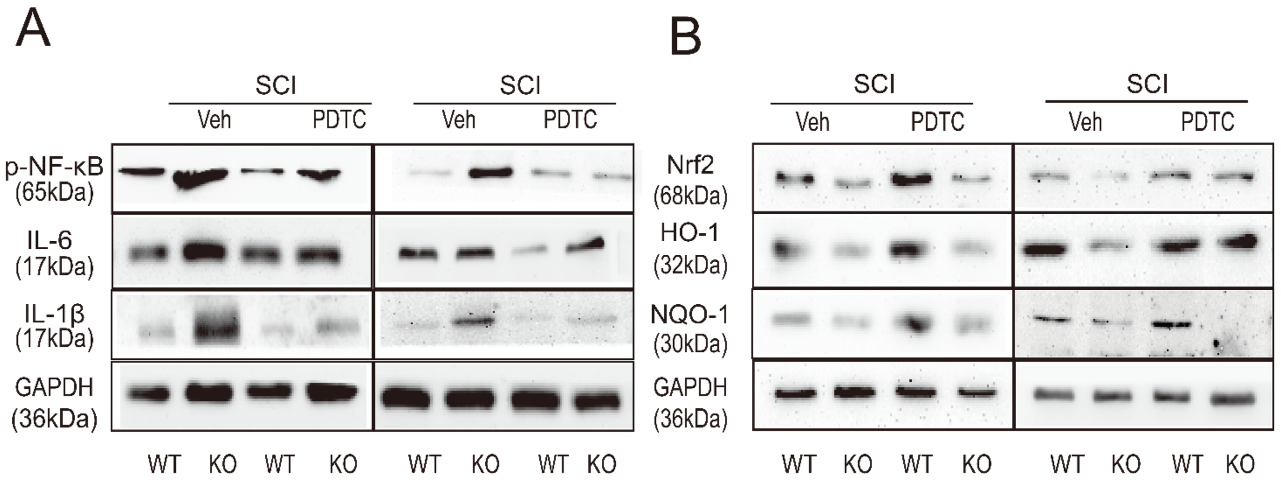


**Supplementary Figure 7: *Apoe* KO mice increased proinflammatory cytokines compared with WT mice after SCI with administration of a NF-κB-inhibitors**

**(A)** Representative Western blots for p-NF-κB, IL-6 and IL-1β expression in spinal cord in WT and *Apoe* KO mice administration with PDTC after 7 days SCI. (n = 6/group).
**(B)** Representative Western blots for Nrf2, HO-1 and NQO1 expression in spinal cord in WT and *Apoe* KO mice administration wit PDTC after 7 days SCI. (n = 6/group).

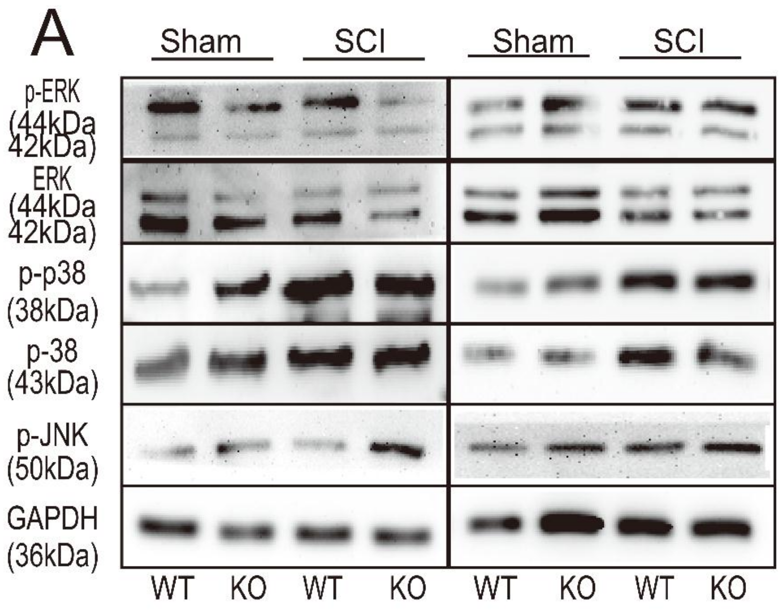


**Supplementary Figure 8: Increased MAPK signal pathway expression in WT mice during the early stage after injury, while *Apoe* deficiency has no apparent effect on these proteins.**

**(A)** Representative Western blots for p-ERK, p-p38, p-JNK expression in spinal cord in WT and *Apoe* KO mice after 7 days SCI. (n = 6/group).
